# Assessing the public perceptions of treated wastewater reuse: opportunities and implications for urban communities in developing countries

**DOI:** 10.1016/j.heliyon.2020.e05246

**Published:** 2020-10-14

**Authors:** Victor E. Akpan, David O. Omole, Daniel E. Bassey

**Affiliations:** Department of Civil Engineering, College of Engineering, Covenant University, P.M.B., Ota, 112233, Nigeria

**Keywords:** Environmental science, Public perception, Wastewater, Wastewater reuse, Recycling, Treatment, Urban communities

## Abstract

Wastewater reuse has become an integral part of Integrated Water Resources Management and thus plays a role in securing the water needs for future generations. This study aimed at determining the perceptions of Canaanland, an emerging urban community in Ogun State, Nigeria, on treated wastewater reuse for several purposes. Data were collected through questionnaires administered to the city residents (n = 244). Findings revealed that the city was aware of the economic and environmental benefits of wastewater reuse but would prefer reuse schemes that involved less human contact such as flushing toilets, electricity generation, building construction, and car wash. The least preferred option was for potable purposes. The community also revealed that they would be willing to accept wastewater reuse as long as it is endorsed by medical doctors, university professors, and experts. However, 45.5% of the respondents were from the Covenant University academic environment. Also, an assessment was carried out to ascertain the implications and opportunities for wastewater reuse in the city. Findings indicated that wastewater reuse involves several complexities and interlinkages, which revolve around political and decisional factors, economic and social factors, environmental factors, and technological factors. From the study, policy and decisional suggestions and a wastewater process flow were developed for more efficient wastewater management within developing cities. A study was carried out on eight cities from developing nations that have created a framework for wastewater management using several approaches. Also, a summary of findings reveals that if adequately researched, cheap and alternative means of wastewater treatment and reuse could be developed for electricity generation, carwash, and firefighting for developing nations. The result of this research can be used to address public anxieties regarding wastewater-reuse practices. Additionally, this study hopes to aid successful wastewater management schemes in the foreseeable future.

## 1 Introduction

Access to clean water and sanitation are some of the enduring challenges faced by humanity. The problem of access to water and sanitation is thus significant enough to constitute one of the Sustainable Development Goals (SDG) number 6, which aims to ensure the availability and sustainable management of water and sanitation for all (United Nations, 2020). Poor sanitation practices are contributors to reduced water quality, while inadequate water supply also impedes access to sanitation. For instance, the leading cause of human mortality globally has been traced to water-related diseases, and more than half of all hospitalized people in Africa were reported to be suffering from water or sanitation-related diseases (Moe and Rheingans, 2006; Omole and Ndambuki, 2014). Rural areas are said to be more prone to sanitation-related illnesses as women and children are reported to spend most of their productive hours daily fetching water from potentially hazardous sources (Omole and Ndambuki, 2014; UN-Women, 2020). Poor access to both water and sanitation impacts on quality of life (Sgroi et al., 2018). Furthermore, as a result of rapid urbanization, population growth, climate change, desertification, and the uneven distribution of water resources in some parts of the world, water demand has increasingly outgrown its supply. These challenges have plunged the world into one form of water crisis or the other (Gain and Wada, 2014; Ren et al., 2017; Schwabe et al., 2020; Sgroi et al., 2018). In some parts of the world, these scarcity challenges are even more visible when compared to other regions. For example, in China, pollution problems are seen to exacerbate the nation's water problems, coupled with the uneven distribution of its water resources (Ma et al., 2020). In the United States, power production has been reported to be subject to several vulnerabilities because of water scarcity and rising stream temperatures due to climate change (Ganguli et al., 2017). Additionally, about 1.6 billion humans living within 328 country-basin units have to suffer a severe water scarcity crisis for at least one month annually. Also, people living within 175 country-basins suffer 3–12 months of water scarcity annually (Degefu et al., 2018). Kummu et al. (2016), who studied the roadmap of water stress in the 20^th^ century, revealed that the population of people facing water shortages had risen from 0.24 billion in the 1900s to 3.8 billion people in the year 2000. These values represent 14% and 58% of the population in their respective years (Kummu et al., 2016).

Sustainable development is significantly hampered as a result of water scarcity (Cheng et al., 2009; Jiang, 2009, 2015; Ma et al., 2020). In the business world, water shortages are reported to affect the operations and supply chains of businesses across the globe. 22% of water consumption globally relates to the production of goods. Some countries, such as the USA, Argentina, Brazil, and Australia, rely on domestic water sources. In contrast, nations like Japan, Mexico, and some European countries depend on water imports to supplement the water supply for industrial purposes. This implies that a shortage of water resources can affect world populations directly or indirectly (Hoekstra, 2014). In a developing country like Nigeria, access to potable water supply has been significantly influenced by factors such as poverty, uneven distribution of water resources, and others. For instance, Abubakar (2019) revealed that 78% of homes in the Northwest and Northeast regions of Nigeria rely on unprotected wells for potable water supply. Also, the high cost of water supply, coupled with the national minimum wage, has led to low access to potable water supply in Yenogoa, Nigeria (Ohwo and Abotutu, 2014). Also, a study by Mary (2014) in Osiele, Ogun state, Nigeria, revealed that most of the residents had limited access to clean water (less than 100 L of water a day). Also, the study showed that the water supply wells in the area had poor sanitary features. Omole (2013) revealed that due to the inadequate provision of water by governmental agencies, over 162.5 million people in Nigeria result in indiscriminate groundwater abstractions for their daily water needs. These rising issues have put humans in search of alternative water sources to supplement their daily needs. Policymakers around the world are considering treated wastewater reuse (WWR) as a suitable alternative to supplement freshwater resources. In 2000, the Millennium Development Goals (MDGs) planned for lessening the number of the global population living without practical access to safe drinking water and sanitation by 2015. This goal, however, did not consider water quality or wastewater management perspectives (Tortajada, 2020). This oversight has been redressed, however, in the SDGs formed on the 1^st^ of June 2012, where one of the objectives (SDG 6) calls for clean water and sanitation for all individuals (Bain et al., 2012; Tortajada, 2020; United Nations, 2020).

WWR has become an integral part of the Integrated Water Resources Management (IWRM) (Yue et al., 2017). Therefore, its importance cannot be overemphasized. Several studies have outlined the importance of WWR in the areas of agriculture, industry, urban development, domestic reuse, potable water supply, and others. In the United States, for example, metropolitan treated WWR, which provides horticultural and urban irrigation system, industrial processes, and potable water use, is reported to have expanded from 4 million m^3^ per day in 1995 to almost 8.5 million m^3^ per day in 2015 (Schwabe et al., 2020). Its benefits, especially in agriculture, has been highlighted by several studies ranging from sufficient nutrients, fertilizer alternatives, prevention of water pollution, increased energy savings, increased food production amongst others (Corcoran et al., 2010; FAO, 2015; Jaramillo and Restrepo, 2017; Omole et al., 2019; Qadir et al., 2010).

The benefits attributed to WWR are subject to its public acceptance within any given society, hence the need for perception studies. Resistance from the public on WWR projects have accounted for failures in many reuse projects (Adewumi et al., 2014). As such, policy and decision-makers need to undertake comprehensive perception appraisals on proposed areas before venturing into treatment and WWR projects to increase the likelihood of success. Several studies have investigated the public perceptions of treated WWR and have come up with different findings. These findings are usually based on the study location and, in many cases, the demographic information of the considered community or nation. For instance, in the United States, Garcia-Cuerva et al. (2016) revealed that a general disgust on the reuse of reclaimed wastewater "yuck factor" was the main challenge facing the acceptability of treated WWR schemes in the country. However, their study revealed that financial incentives influenced the general public decision on reusing treated wastewater. Similarly, negative emotional reactions "yuck factor" to the reuse of treated wastewater were recorded by Wester et al. (2015). Both studies revealed that women who possessed lower levels of education were more susceptible to discomfort regarding treated WWR. Also Garcia-Cuerva et al. (2016) revealed that respondents in the United States were willing to reuse treated wastewater so long it was not deployed for the cultivation of food crops. However, a study conducted in the southeastern region of Italy revealed different situations, as a high percentage of the residents, especially farmers, demonstrated a willingness to reuse treated wastewater for agricultural purposes (Saliba et al., 2018). Similarly, a perception study in Tunisia and Jordan showed that farmers and residents had high levels of acceptance to use reclaimed wastewater to food products (Abu-Madi et al., 2008a). However, an Israel based study revealed that only 49% of the respondents were in support of reusing treated wastewater for orchard irrigation. Furthermore, the study revealed that 95% of the respondents were in support of reusing treated wastewater for sidewalk irrigation, 96% for firefighting, 85% for flushing toilets, and 62% for aquifer recharge. The least preferred reuse scheme was potable aquifer recharge, as only 11% of the respondents were in support of it (Friedler et al., 2006). Also, respondents in another study revealed that respondents were in support of treated WWR for just non-potable purposes (Oteng-Peprah et al., 2018).

A comparison of the de facto WWR in three cities (Phoenix, AZ, Atlanta, GA, Philadelphia, PA) in the United States revealed that 25% of respondents perceive de facto reuse to occur in their home tap water. These set of people who perceived de facto reuse to happen at their taps were ten times more likely to have a high level of acceptance (Rice et al., 2016). In the case of Australia, Dolnicar & Schäfer (2009) revealed that a five year drought period accompanied by strict water restrictions and subsequent media attention to water scarcity solutions now make Australians more receptive to reusing treated wastewater for garden watering and cleaning uses. The acceptability of treated WWR was, however, very poor in Australia in the past. In the Palestinian territory Deir Debwan, general water shortages have forced the residents to source for alternative water supply. A study conducted by Abu-Madi et al., 2008b revealed that 87% of the respondents were willing to use treated wastewater, while 85% were willing to consume products irrigated with treated wastewater. The high acceptability of recycled wastewater for agricultural purposes in the Palestinian region is an indicator of the limited water resources in the region. For instance, 70% of water needs in the West Bank region are used for agricultural production; thus, the scarcity in the region pushes residents to embrace other water alternatives such as treated WWR (McNeill et al., 2009). In Tanzania, community members who practice irrigated agriculture using effluent discharge from their homes were in support of treated WWR (Kihila et al., 2014). The reuse of treated wastewater receives wide acceptance as a means for responsible water resources management as long as close contact with humans is minimal (Nancarrow et al., 2008). A study by Adewumi et al. (2010) in South Africa revealed that there was a significant potential for implementing treated WWR for large non-potable applications such as landscape irrigation and industrial processes, especially in the arid regions of the country.

**Figure 1:**
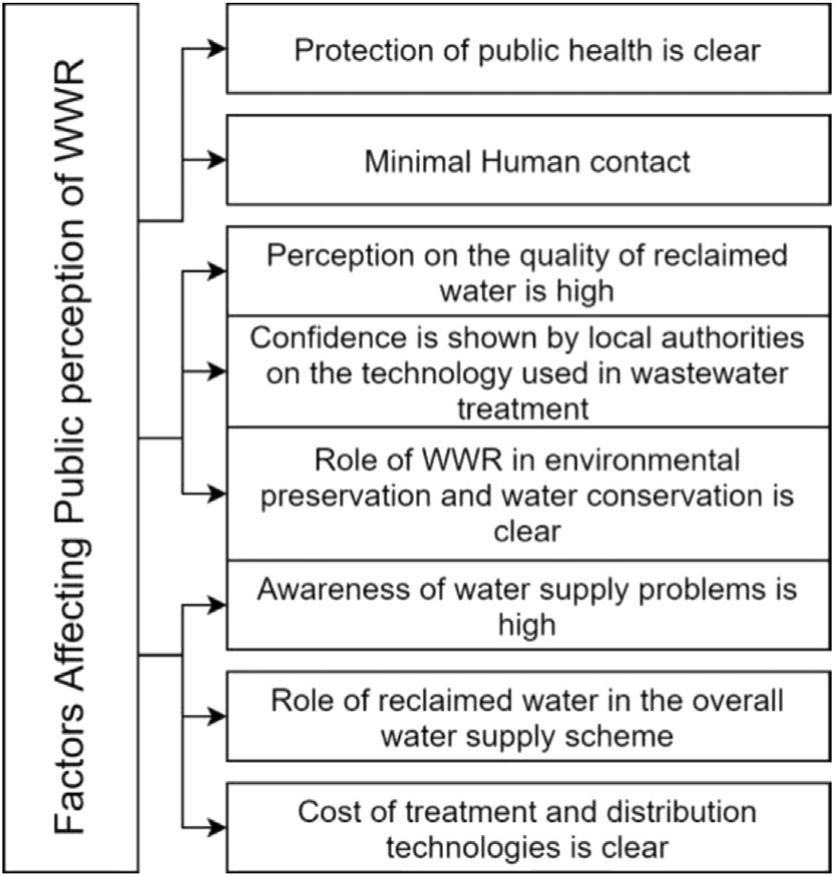
Reoccurring factors that affect the public perception on reusing treated wastewater.

However, the reuse of treated wastewater has raised several concerns. For instance, a study by Buyukkamaci and Alkan (2013) in Turkey revealed that both men and women had raised concerns over the health risks associated with the reuse of treated wastewater, especially for potable purposes. Respondents identified concerns about the imminent threat of water-borne diseases in Beirut, Lebanon. The study revealed a lack of trust for treated WWR due to public health concerns (Massoud et al., 2018). Also, the study revealed that the public was also concerned with the reclamation costs involved with reusing treated wastewater. Different responses were seen in the United States as two-thirds of the respondents in a study conducted in Arizona revealed that they were willing to support increasing water or sewer rates to treat water to higher standards (Rock et al., 2012). In the Arizona study, the level of education had a significant impact on the level of treated WWR acceptability. However, in Tucson, Arizona, the public acceptability of treated WWR is contingent on trust in the authorities who influence the design of sociotechnical systems such as water and wastewater utilities, consultants, academics, local officials, and regulators (Ormerod and Scott, 2013). A similar finding in Queensland indicated that community members were more receptive of treated WWR as long as they perceived that the water authority used fair procedures such as consulting and adequate dissemination of information to the public (Ross et al., 2014). Figure 1 gives a summary of the common reoccurring factors that affect the public perceptions of reusing treated wastewater from literature.

The reuse of treated wastewater, no matter how beneficial, could lead to consequences if not approached or managed efficiently. Several studies have indicated the potential hazards that could result from WWR, especially when careful planning and execution have not been put in place. For instance, Delli Compagni et al. (2020) developed an integrated model to predict the fate of Contaminants of Emerging Concern (CECs) in water reuse systems that utilized reclaimed wastewater for cultivation of edible crops. The findings from their study revealed that customers faced a health risk from sulfamethoxazole and 17α-ethinylestradiol. Similarly, a study carried out by (Alygizakis et al., 2020) to determine CECs from a wastewater treatment plant in Cyprus revealed that six antibiotic-resistant genes were detected in all collected wastewater samples. Therefore, the importance of critical treatment evaluation on a routine basis cannot be overemphasized.

This study is focused on understanding the public perception of treated WWR to highlight hidden opportunities and implications for emerging cities in developing countries considering the scenario of Canaanland, Nigeria. Canaanland, Nigeria, was selected for this survey due to the willingness of the city to adopt alternative water resources to supplement its ever-growing water demand, and in turn, reduce the extent of groundwater abstraction within the community. Information on public perception of wastewater treatment and reuse in the study location has not been available in previous years. Therefore, this study bridges the data gap of the region on perceptions of reusing treated wastewater for several purposes. Furthermore, there is little literature on the public perceptions of treated WWR in Nigeria, most of which did not account for endorsements that are necessary to convince the potential customers on the reuse of treated wastewater. Additionally, the study proposes a wastewater treatment flow process for developing cities and highlights the roadmaps created by some cities in developing countries using existing technologies.

## 2 Materials and methods

### 2.1 Description of study area

Canaanland is a modern city developed in Ota, Ogun State, Nigeria. It is situated roughly 16km from Lagos, which is a significant economic and financial center for all of Africa (Cobbett, 2018; Ilesanmi, 2010; Mogaji, 2020). It is located at the coordinates 6°40′34°N 3°09′50°E and is home to Covenant University, and one of the Largest church auditoriums in the world. The city is home to approximately 13,000 people and receives over 200,000 weekly visitors (Isiorho et al., 2014). The city has witnessed a rapid expansion and a population boom over the last decade, from about 7000 residents in 2010 to an estimated population of 13000 in 2020. This rapid population boom has led to a significant rise in water consumption, thus increasing the volume of wastewater generated. A study by Omole et al. (2019) revealed that an estimated 1.5 million liters of wastewater discharged from the city's wastewater treatment plant daily flows into the nearby River Atuwara. This has led to reports of fecal contamination in the downstream regions of a nearby river, according to previous studies (Omole et al., 2018, 2019). The city relies on groundwater abstraction for all of its domestic and potable water supply. The city has been reported to pump over 2 million liters of water daily, and the City's Department of Physical Planning and Development (PPD) report that about 30 boreholes continuously work for 24 h to ensure the city never runs out of water (Omole et al., 2019). The PPD is tasked with the responsibility of water supply and wastewater treatment within the Canaanland community.

**Figure 2:**
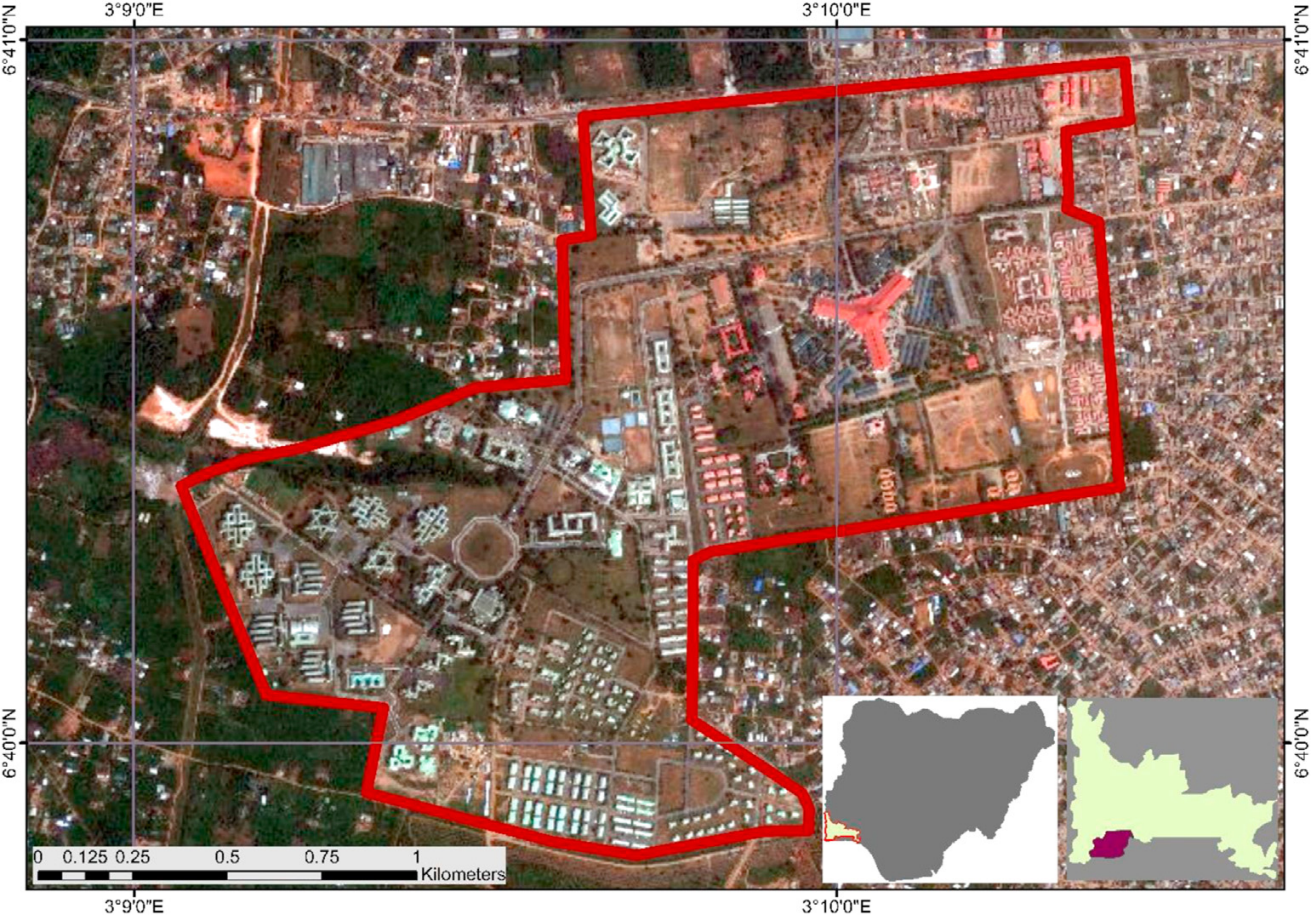
Aerial view of study location with Ogun State and Nigeria: Developed with ESRI® ArcMap 10.7.

Figure 2 shows a map of the study area with Ogun State and Nigeria.

### 2.2 Data collection and analysis

The community's perception of wastewater treatment and reuse for different applications were collected via a quantitative survey. An intentional sample of 320 participants, mostly from the academic environment, was recruited. However, of the 320 questionnaires that were distributed, 244 were returned. This response represents 76% of the total administered surveys. The questionnaire was adapted from structured surveys used by Baawain et al. (2020) and Bungu (2014), and was modified to suit the situation of the study location. For detailed information on the administered questions, see the supplementary material. Based on the theoretical foundations from previous studies, the following hypotheses were formulated about the concerns, acceptability of treated WWR, and general knowledge on treated WWR: (i) education will significantly influence the degree of acceptability of the proposed reuse projects in the Canaanland region, (ii) the knowledge level on global water shortages will be higher in Canaanland given that most of the residents are from the academic area (iii) non-contact reuse projects will be mostly accepted in the Canaanland community. The questionnaire was designed to tackle the following subjects:

i. The demographic information of members of the city, such as age, gender, etc.
ii. Knowledge of the community on wastewater generation within the city
iii. The reason why wastewater reuse may or may not be accepted within the community
iv. The most acceptable wastewater reuse projects within the community
v. How professional endorsements could affect the perceptions of wastewater reuse.

The questions were further divided into three sections to ease the interpretation of responses and also best determine the views of the respondents. Table 1 gives a summary of the different sections.

**Table 1:** Summary of questionnaire sections.

Section	Questions	Group	Answer Type
A	General Views/Perception of wastewater Recycling and Reuse	(Q1-Q9)	Strongly Agree/Agree/Neutral/Disagree/Strongly Disagree
B	Knowledge of Community Water and Wastewater situation	(Q10-Q12)	Yes or No
C	Specific Responses on Reuse, Concerns, and Preference	(Q13-Q19)	Multiple Picks

The study was conducted from January 2020 to March 2020 to determine the public perceptions of the community on the reuse of treated wastewater for specific applications such as firefighting, industrial applications, washing clothes, cooking, electricity generation, potable purposes, swimming pool, and others. The questionnaires were targeted at random individuals within the Canaanland community. Also, the questionnaire was designed in the English language, which is the official language of communication in Nigeria. Five (5) people were selected to administer the questionnaires, all holding a degree in Civil Engineering. The surveyors were briefed with the study objectives to enable them to guide the respondents were necessary (e.g., terminologies used in the questionnaire), and also to retrieve the filled questionnaires. Each questionnaire was reviewed by an experienced field supervisor (a Civil Engineering professor) and was corrected together with the surveyors. Various analytical procedures were applied, including reliability assessments, statistical correlations, statistical percentages, and frequencies. The collected data was cataloged into SPSS (version 25), and Microsoft Excel for further analysis and descriptive statistics were also developed. The *T*-test was used to study the relationship between gender and the reuse of treated wastewater for different purposes. The Chi-square test was used to study the relationship between age, educational qualification, and level of employment on the degree of acceptability of treated WWR for several applications. The Chi-square test was also used to analyze the relationship between age, educational qualification, and level of employment on potential concerns that may prevent the public from reusing treated wastewater. Also, the internal consistency of the questionnaire was determined using Cronbach's alpha coefficient, which is expressed with a number between 0 and 1. Reliability simply means how well a test measures what it is designed to measure. Cronbach's Alpha can be written as a function of the number of test items and the average inter-correlation among the items (UCLA, 2019). The SPSS software was used to assess the reliability of the questionnaire. The closer the reliability value is to 1, the less error there is. For example, the reliability of 0.8 indicates that the variables are 80% true with a 20% error. The equation for the Cronbach's Alpha is given in Eq. (1)

(1) $$\alpha =\frac{N\bar{c}}{\bar{v}+\left(N-1\right)\bar{c}}$$

Where: α = Cronbach's alpha, N = number of items, $\bar{c}$= the average inter-item covariance, and $\bar{v}$ = average variance.

### 2.3 Demographic data

From the 244 respondents to the survey, 147 (60.2%) were males, while 97 (39.8%) were females. The highest fraction of the respondents were in the 21–30 age group, representing 75.8% of the total number of respondents. This was followed by respondents between ages 16–21 (12.7%). Other age groups were 31–40, 41–50 and 50+, at 7.4%, 3.3% and 0.8%, respectively. 46.3% of the respondents were BSc holders, while 25% of the respondents were master's degree holders. This BSc group represented the highest level of respondents based on the degree of education. Undergraduate students comprised of 18.4% of the respondents. Respondents with diplomas and Ph.D. comprised 3.7% each of the total respondents, respectively. 2.4% of the respondents were high school graduates, while 0.4% had no level of education. The majority of respondents were comprised mainly of students at 45.5%. 31.1% of the respondents reported being employed within the city, while 16.4% were private business owners. 7% of the respondents had no form of employment. Table 2 gives a summary of the demographic information of the respondents.

**Table 2:** Demographic information of attendees.

Category	n	Distribution (%)
Gender		
Male	147	60.2
Female	97	39.8
Age Group		
16–21	31	12.7
21–30	185	75.8
31–40	18	7.4
41–50	8	3.3
51>	2	0.8
Level of Qualification		
No level of Education	1	0.4
Secondary School	6	2.5
Undergraduate Students (Enrolled in a BSc Program)	45	18.4
Diploma (OND/HND)	9	3.7
BSc Graduates	113	46.3
Masters Degree	61	25
PhD	9	3.7
Employment Level		
Student	111	45.5
Self Employed (Owners of Private Businesses)	40	16.4
Employed	76	31.1
Unemployed	17	7

## 3 Results

### 3.1 Background information on wastewater recycling and reuse

**Figure 3:**
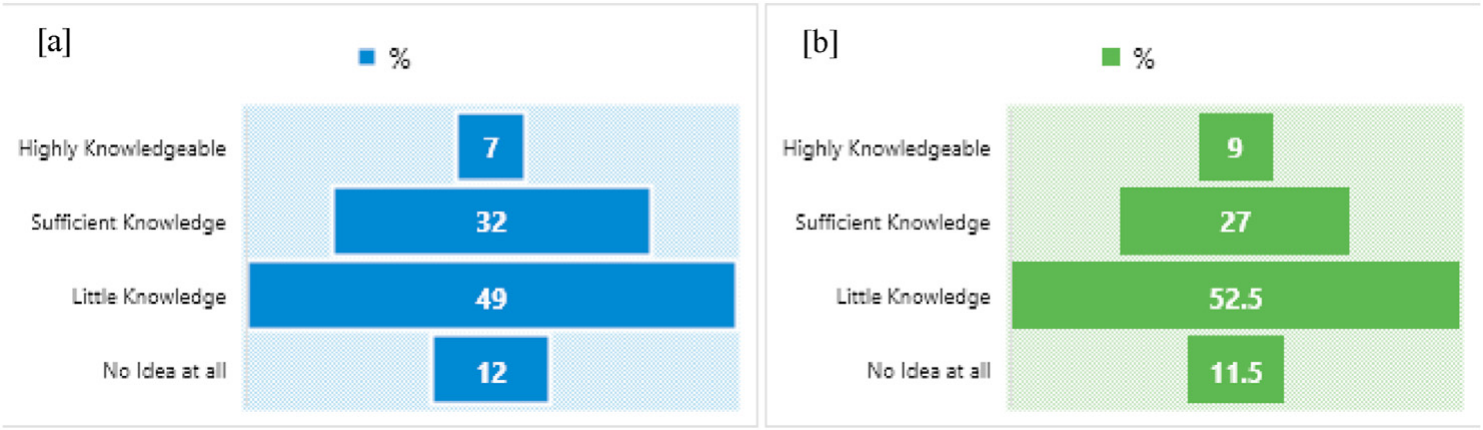
(a) respondents' knowledge of wastewater recycling and reuse (b) statistics on the knowledge of global water shortages.

From the questionnaires received, 128 (52.5%) respondents had little knowledge of water shortages in some regions of the world. This was followed by 66 (27%) respondents with sufficient knowledge. 22 (9%) respondents were highly knowledgeable, while 28 (11.5%) had no idea at all on global water shortages in some regions of the world. Figure 3a gives statistics on the knowledge of global water shortages. Furthermore, the knowledge of the population on wastewater recycling revealed that 120 (49.2%) the respondents had little knowledge about wastewater recycling and reuse. 78 (32%) respondents had a sufficient understanding of wastewater recycling and reuse, while 29 (11.9%) had no idea at all. 7% of the respondents were highly knowledgeable about the subject of wastewater recycling and reuse. Figure 3b shows the respondents' knowledge of wastewater recycling and reuse.

### 3.2 Reliability assessment

The Cronbach's alpha coefficients for this study are given in Table 3.

**Table 3:** Cronbach's alpha coefficients for the study.

Q		Cronbach's Alpha	Cronbach's Alpha Based on Standardized Items	Number of Items	Cronbach's Alpha if the item is Deleted
	**Section A**				
Q1	Recycling and reusing wastewater is environmentally responsible	0.905	0.874	9.00	0.886
Q2	Recycling and Reusing wastewater protects the environment from pollutants				0.88
Q3	Recycling and Reusing Wastewater could cause health concerns				0.938
Q4	Reusing wastewater will reduce the need for water treatment plant expansions				0.932
Q5	Recycling and Reusing wastewater will bring about economic benefits				0.875
Q6	Recycled wastewater can serve as a source of Fertilizer				0.879
Q7	Wastewater reused in agriculture can boost agricultural yield				0.872
Q8	Energy-saving is a potential of wastewater reuse				0.870
Q9	Money can be made from wastewater reuse				0.888

	**Section B**				
Q10	Have you seen a wastewater treatment facility?	0.602	0.745	3	0.896
Q11	Does your community or Institution have a wastewater treatment facility?				0.121
Q12	Do you think groundwater levels in your community is sustainable at the current pumping rate?				0.57

From Table 3, the reliability assessment revealed a Cronbach's Alpha of 0.905 for the questions in section A and 0.602 for section B, which is acceptable given the acceptability criteria of 0.50 (Bungu, 2014). For section A, the construct showed an error level of 5% and 95% correctness for the variables, which is highly acceptable. Section B showed a much lower internal consistency at 0.602, which is much lower than the 0.70 recommendation by experts. However, this study determines ability, and thus 0.50 and above are acceptable (Bungu, 2014).

### 3.3 Descriptive statistics

This section gives results to specific responses of the respondents and highlights the user's preferences and concerns.

#### 3.3.1 General Views/Perception of wastewater recycling and reuse

Table 4 contains responses for section A and B, respectively.

**Table 4:** General Views/Perception of wastewater Recycling and Reuse.

**Q**	**Section A**	**Strongly Disagree (1)**	**Disagree (2)**	**Neutral (3)**	**Agree (4)**	**Strongly Agree (5)**	**Completed**	**Missing**	**Mean**	**STDEV**
Q1	Recycling, treating, and reusing wastewater is environmentally responsible	8	10	39	93	94	244	0	4.05	3.65
Q2	Recycling, treating, and reusing wastewater protects the environment from pollutants	14	11	31	110	77	243	1	3.93	3.553
Q3	Recycling, treating, and reusing wastewater could cause health concerns	34	82	70	47	11	244	0	2.67	2.368
Q4	Reusing treated wastewater will reduce the need for water treatment plant expansions	37	70	75	51	9	242	2	2.69	2.39
Q5	Recycling, treating, and Reusing wastewater will bring about economic benefits	6	7	30	139	60	242	2	3.99	3.557
Q6	Recycled wastewater can serve as a source of Fertilizer	8	12	77	89	50	236	8	3.68	3.29
Q7	Treated wastewater reused in agriculture can boost agricultural yield	7	12	72	108	44	243	1	3.7	3.291
Q8	Energy-saving is a potential of treated wastewater reuse	5	9	69	121	39	243	1	3.74	3.311
Q9	Money can be made from treated wastewater reuse	2	3	45	90	100	241	3	4.18	3.739
	**Section B**	**No**	**Unsure**	**Yes**						
Q10	Have you seen a wastewater treatment facility?	122	22	90			234	10	1.86	1.58
Q11	Does your community or Institution have a wastewater treatment facility?	106	74	61			241	3	1.81	1.46
Q12	Do you think groundwater levels in your community is sustainable at the current pumping rate?	108	89	40			237	7	1.95	1.57

**Figure 4:**
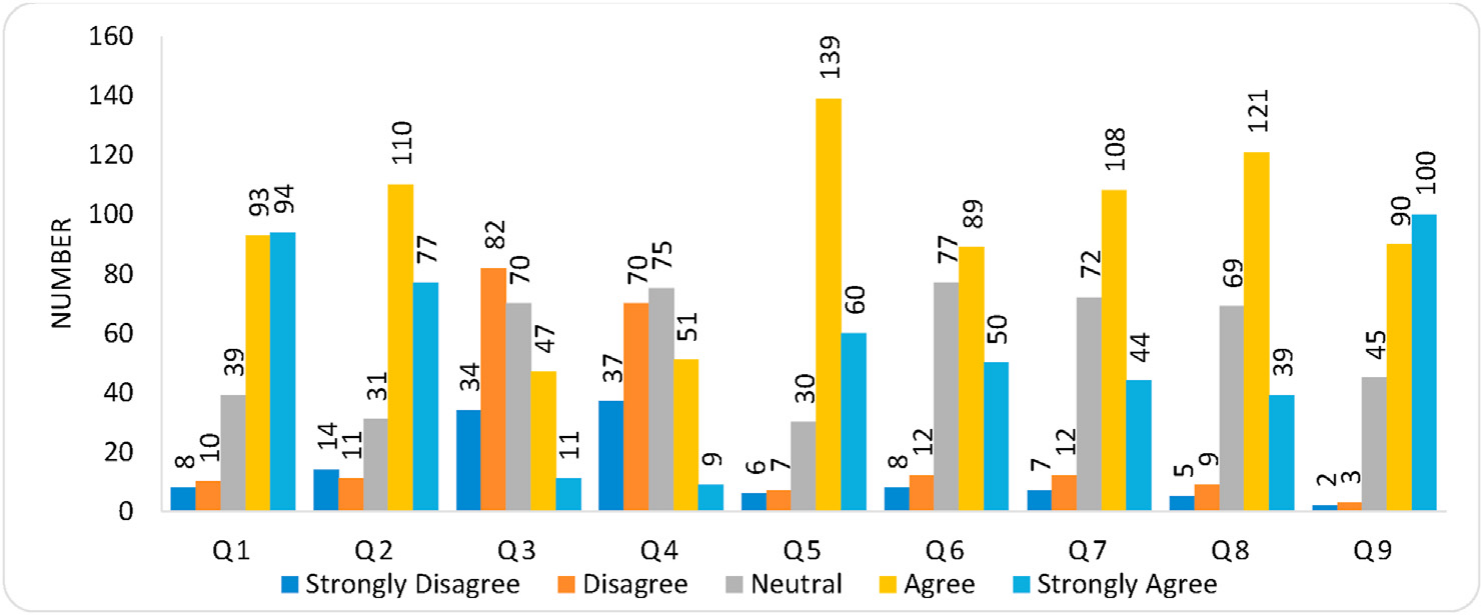
Responses to General Views/Perception of wastewater Recycling and Reuse (Section A).

From Table 4, about 77% of respondents agree that recycling and reusing wastewater will be an environmentally responsible decision to be taken in the community. Also, 77% of respondents agree that recycling and reusing treated wastewater will protect the environment from pollutants. 47.5% of respondents disagree that a well-managed wastewater treatment and reuse scheme could pose health hazards. However, 23.8% have concerns about the health implications of reusing treated wastewater, and 28.7% of the respondents were neutral on the health implications of recycling and reusing wastewater. This implies that a lot of efforts must go into professional endorsements and quality checks to convince the university community that the reuse process will be safe. Additionally, 82% of respondents believe that wastewater recycling and reuse are economically profitable. 60% of respondents see treated wastewater as a good source of fertilizer for watering lawns, farms, and others. Also, 63% agree that treated WWR could boost agricultural productivity. 66% of correspondents agree that energy saving is the potential for treated wastewater reuse. Figure 4 gives a graphical representation of responses to the questions in section A in Table 4.

**Figure 5:**
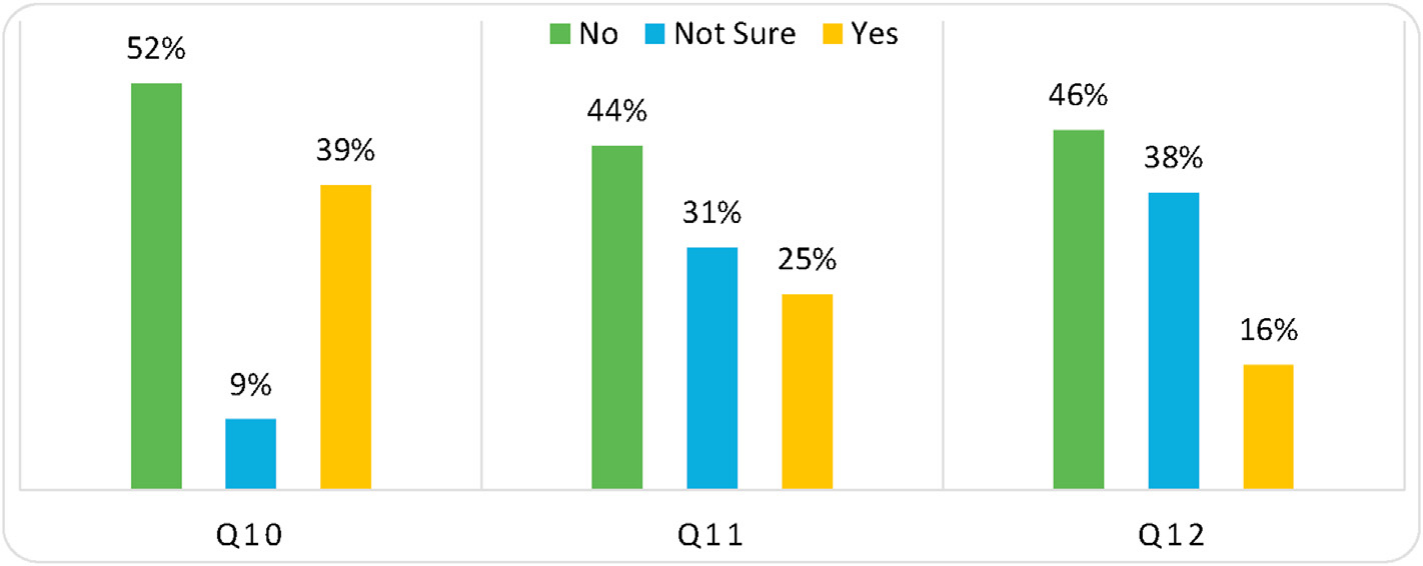
Responses to Knowledge of Community Water and Wastewater situation.

Furthermore, section B revealed that 61% of the respondents have neither seen a wastewater treatment plant or not sure if they had seen any treatment facility. 75% of the respondents were not aware of the Institution's wastewater treatment facility. However, only 16% of the respondents agreed that the groundwater level in the community is sustainable, given the current pumping rate. This figure leaves 84% of the respondents either not sure of the present pumping rate or with concerns over the current pumping rate within the university community. Figure 5 gives a graphical representation of the responses to section B of the survey.

#### 3.3.2 Specific Responses on Reuse, concerns, and preference

**Figure 6:**
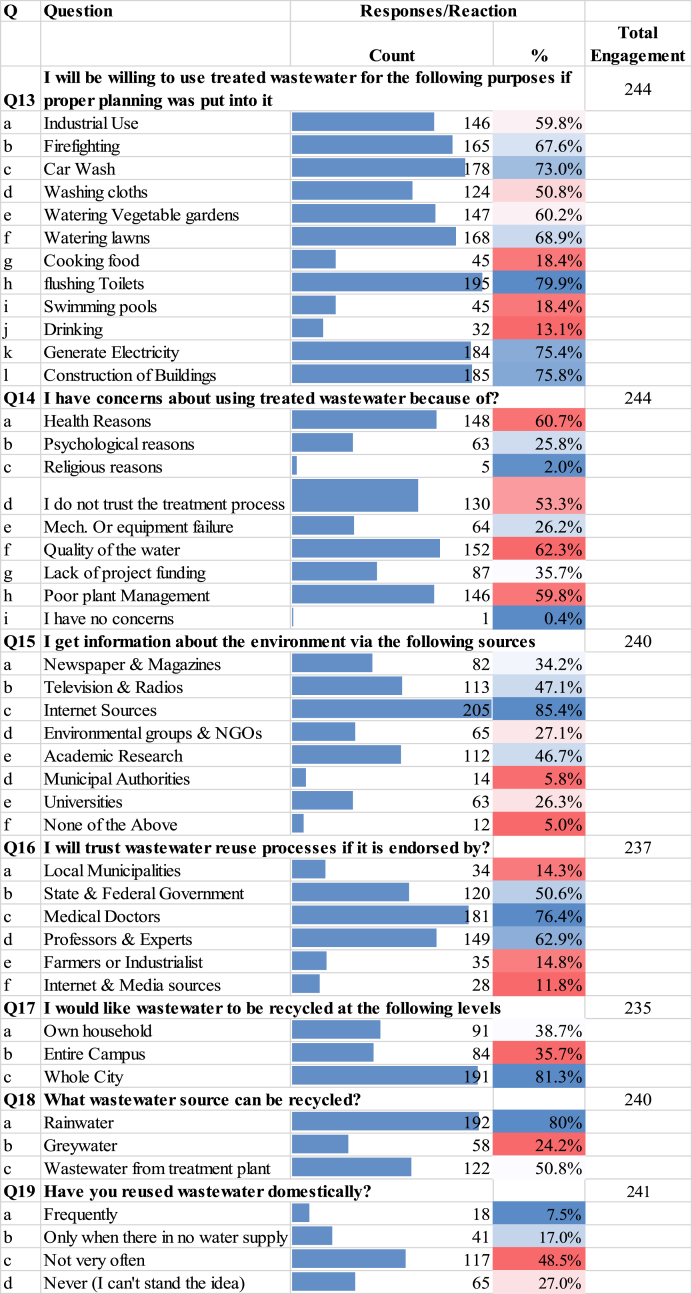
Specific responses on reuse, concerns, and preference.

Figure 6 highlights specific responses of the public on preferences for reuse as well as concerns and the required professional endorsements that could aid the acceptance of WWR within the community. Bar charts and color codes are embedded to interpret the data best. Colors with red signify a less favorable option, while blue implies a more preferred choice.

From Figure 6, Q13 reveals that most respondents were willing to utilize treated wastewater for activities such as flushing toilet (80%), building and other construction processes (76%), generating Electricity (75.4%), car wash (73%), watering lawns (69%), and firefighting (67.6%). The levels of acceptance were seen to decrease as human contact increases. The least acceptable reuse schemes were cooking food (18.4%), swimming pools (18.4%), and drinking (13.1%).

Q14 identifies the community's concerns on treated wastewater reuse. 62.3% of the respondents were concerned about the quality of treated wastewater for reuse, followed by concerns on health grounds at 60.7%. 59.8% of respondents were worried about poor plant management, with 53.3% raising concerns for their lack of trust for the potential treatment processes. The least causes of concerns were psychological reasons and religious reasons at 25.8% and 2% respectively.

Q15 reveals that a majority of the respondents (85.4%) get information regarding environmental issues from internet sources. This information source was trailed by television and radios at 47.1% and academic research at 46.7%. The least source of information were universities and municipalities at 26.3% and 5.8%, respectively.

Q16 reveals how professional endorsements will affect the treated wastewater reuse process. 76.4% of the respondents will only trust the wastewater recycling, treatment, and reuse process if medical professionals endorse it. 63% of the respondents would trust the process when it is recommended by professors and experts in the field of wastewater reuse. 50.6% of the respondents will trust the reuse process when state and federal governments give the go-ahead. The least trusted endorsements, according to respondents, would come from farmers and industrialists (14.8%), local municipalities (14.3%), and internet and media sources (11.8%).

Q17 shows that 81.3% of the respondents would prefer wastewater to be recycled at the city or municipal level. Q18 revealed that 80% of the respondents would prefer rainwater recycling as the best recycling option, while 50.8% of the respondents preferred the reuse of treated wastewater from the treatment plants.

Q19 reveals that only 7.5% of the respondents frequently reuse wastewater domestically. 17% of respondents reuse wastewater locally only in the advent of water shortages. 27% of respondents reveal that they have not reused wastewater domestically and cannot stand the idea of wastewater reuse at domestic levels.

### 3.4 Influence of demographic variables on treated wastewater acceptability and potential concerns

#### 3.4.1 Influence of educational qualification on treated WWR applications and potential concerns

Past studies have revealed that educational and locational factors could influence the acceptability of treated wastewater for several purposes (Mu'azu et al., 2020). In the present Canaanland investigations, the results of Chi-square (χ^2^) analysis in Table 5 indicates that there was no statistically significant (SS) difference (*p*-values > .1) between the respondents level of education and the choice of acceptability on treated WWR for most of the selected reuse projects with χ^2^ values ranging between 2.619 and 16.382. The exceptional cases of significant difference between the respondents educational qualifications and degree of acceptability for the chosen reuse projects were for watering lawns (p = .037 and χ^2^ = 13.438), cooking food (p = 0.030 and χ^2^ = 13.945), for potable purposes (p = 0.012 and χ^2^ = 16.382), and swimming pools (p = 0.070 and χ^2^ = 11.676). Particularly, the reuse of treated wastewater for potable purposes showed the highest significant level, which was not unexpected, considering that it involves direct consumption. The insignificant p-values can be attributed to the bulk of the respondents residing in the academic area of the city. Also, only 0.4% of the respondents declared that they had not received any form of formal education.

**Table 5:** Influence of educational qualification on treated WWR applications and possible concerns.

Variables	χ^2^	Degree of freedom	Significance (p-value)
Relationship between Educational Qualification and Reuse Application			
Industrial use	4.569	6	0.600
Firefighting	8.982	6	0.175
Washing cars	4.526	6	0.606
Washing clothes	4.789	6	0.571
Watering vegetables	5.795	6	0.447
Watering lawns	13.438	6	0.037
Cooking food	13.945	6	0.030
Flushing toilet	5.945	6	0.429
Swimming pools	11.676	6	0.070
Drinking	16.382	6	0.012
Generating electricity	9.320	6	0.156
Construction of buildings	2.619	6	0.855
Relationship between Educational Qualification and potential concerns			
Health reasons	10.646	6	0.100
Psychological reasons	9.853	6	0.131
Religious reasons	24.337	6	<0.001
I do not trust the treatment process	7.656	6	0.264
Mechanical or equipment breakdown	6.613	6	0.358
Quality of water	3.797	6	0.704
Lack of funds for the process	18.850	6	0.004
Poor management of the plant	9.102	6	0.168

Significant at p < .10.

In the area of the public concerns on reusing treated wastewater, the study showed that there were no SS differences for most of the identified concerns with regards to educational qualifications with χ^2^ values ranging between 3.797 and 24.337. However, religious concerns and lack of funds for treatment and reuse of wastewater showed a high degree of SS at (p < 0.001 and χ^2^ = 24.337) and (p = 0.004 and χ^2^ = 18.850) respectively. This proves that the level of education among the respondents had a significant impact on their religious views regarding treated WWR. Table 5 also contains the results of χ^2^ analysis on the relationship between educational qualification and potential concerns for treated WWR.

#### 3.4.2 Influence of age on treated WWR applications and potential concerns

The χ^2^ analysis of the association between the age groups and the level of acceptability of treated WWR revealed that there was an insignificant (*p*-values > .1) relationship for all the proposed reuse projects with χ^2^ values ranging between 1.325 and 6.629. However, previous studies have revealed a significant relationship between the age groups of respondents to the degree of acceptability of treated WWR. Some studies have revealed that older participants were most likely to reject the reuse of treated wastewater when compared to their younger counterparts (Buyukkamaci and Alkan, 2013; Mu'azu et al., 2020). Also, in some studies, the older participants were more likely to accept the reuse of treated wastewater when compared to their younger counterparts (Fielding et al., 2019; Gu et al., 2015). These disparities on the influence of age on treated WWR are reported to be confounded by chronological and spatial divides (Fielding et al., 2019; Mu'azu et al., 2020). In the area of public concerns, there was an insignificant relationship between the ages of the respondents and the potential concerns with χ^2^ values ranging between 1.823 and 4.821. Table 6 gives the results of the χ^2^ analysis on the relationship between age and treated wastewater reuse application. It also presents the relationship between age and the concerns of the population.

**Table 6:** Influence of age on treated WWR applications and potential concerns.

Variables	χ^2^	Degree of freedom	Significance (p-value)
Relationship between Respondents' Age and Reuse Application			
Industrial use	3.214	4	0.523
Firefighting	4.677	4	0.322
Washing cars	6.629	4	0.157
Washing clothes	4.120	4	0.390
Watering vegetables	5.210	4	0.266
Watering lawns	4.638	4	0.327
Cooking food	3.301	4	0.509
Flushing toilet	1.355	4	0.852
Swimming pools	2.774	4	0.596
Drinking	1.691	4	0.792
Generating electricity	1.323	4	0.857
Construction of buildings	1.649	4	0.800
Relationship between Respondents' Age and potential concerns			
Health reasons	2.929	4	0.570
Psychological reasons	1.823	4	0.768
Religious reasons	1.972	4	0.741
I do not trust the treatment process	4.416	4	0.353
Mechanical or equipment breakdown	3.196	4	0.526
Quality of water	4.821	4	0.306
Lack of funds for the process	4.536	4	0.338
Poor management of the plant	2.999	4	0.558

Significant at p < .10.

#### 3.4.3 Influence of employment level on treated WWR applications and potential concerns

The χ^2^ analysis of the association between the level of employment and the level of acceptability of treated WWR revealed that there was an insignificant (*p* > .1) relationship for some of the proposed reuse projects with χ^2^ values ranging between 0.105 and 8.975. The exceptional cases of significant (*p*-values < .1) difference between the respondents level of employment and degree of acceptability for the chosen reuse projects were for firefighting (p = 0.086 and χ^2^ = 6.584), cooking food (p = .030 and χ^2^ = 8.922), for potable purposes (p = 0.034 and χ^2^ = 8.658), swimming pools (p = 0.083 and χ^2^ = 6.666) and generating electricity (p = 0.030 and χ^2^ = 8.975).

In the area of the public concerns on reusing treated wastewater, the study showed that there were no SS (*p*-values > .1) differences for most of the identified concerns with regards to the level of employment of the respondents, with χ^2^ values ranging between 1.028 and 12.518. However, religious concerns showed a high degree of SS at (p = 0.006 and χ^2^ = 12.518). Table 7 contains the χ^2^ results of the analysis on the relationship between employment levels and potential concerns.

**Table 7:** Influence of employment level on treated WWR applications and potential concerns.

Variables	χ^2^	Degree of freedom	Significance (p-value)
Relationship between Respondents' Employment Status and Reuse Application			
Industrial use	0.269	3	0.966
Firefighting	6.584	3	0.086
Washing cars	2.035	3	0.565
Washing clothes	3.142	3	0.370
Watering vegetables	3.554	3	0.314
Watering lawns	0.105	3	0.991
Cooking food	8.922	3	0.030
Flushing toilet	1.324	3	0.723
Swimming pools	6.666	3	0.083
Drinking	8.658	3	0.034
Generating electricity	8.975	3	0.030
Construction of buildings	5.589	3	1.133
Relationship between Respondents' Employment Status and potential concerns			
Health reasons	4.513	3	0.211
Psychological reasons	3.475	3	0.324
Religious reasons	12.518	3	0.006
I do not trust the treatment process	2.898	3	0.408
Mechanical or equipment breakdown	4.545	3	0.208
Quality of water	1.640	3	0.650
Lack of funds for the process	1.529	3	0.676
Poor management of the plant	1.028	3	0.794

Significant at p < .10.

#### 3.4.4 Influence of gender on treated WWR applications

The influence of gender on the acceptability of treated wastewater was determined using the t-test because gender is a dichotomous variable. The results indicate that some of the p-values (washing clothes, watering vegetables, and watering lawns) are >.1, which implies that the opinion on treated WWR for those projects was insignificantly associated with gender. However, the p-values (industrial use, firefighting, washing cars, cooking food, flushing toilet, swimming pools) are <.1, which implies that the opinion on treated WWR for those projects was significantly associated with gender. Many of the recent studies on the influence of gender on accepting treated WWR found no significant relationship, albeit men are more likely to accept more risk-prone options than women (Fielding et al., 2019; Mu'azu et al., 2020). Subsequently, within the Canaanland setting, gender disparity is expected to be a key factor when considering some treated WWR options. The t-test results are presented in Table 8.

**Table 8:** Influence of gender on treated WWR applications.

Independent variable	Demography variable	Descriptive Statistics				Levene's Test for Equality of Variances		
	Gender	N	Mean	Std. Deviation	Std. Error Mean	F	Sig.	t
Industrial Use	Male	147	0.6735	0.47055	0.03881	12.879	<0.001	2.499
	Female	97	0.5155	0.50236	0.05101			2.465
Firefighting	Male	147	0.7211	0.45	0.03712	16.406	<0.001	2.343
	Female	97	0.5773	0.49655	0.05042			2.296
Washing cars	Male	147	0.7687	0.4231	0.0349	12.608	<0.001	1.871
	Female	97	0.6598	0.47624	0.04835			1.826
Washing clothes	Male	147	0.5782	0.49553	0.04087	0.115	0.735	2.236
	Female	97	0.433	0.49806	0.05057			2.234
Watering vegetables	Male	147	0.6122	0.4889	0.04032	0.193	0.661	0.223
	Female	97	0.5979	0.49286	0.05004			0.223
Watering lawns	Male	147	0.6871	0.46527	0.03837	1.415	0.235	0.61
	Female	97	0.6495	0.47961	0.0487			0.606
Cooking food	Male	147	0.2517	0.43547	0.03592	60.117	<0.001	3.4
	Female	97	0.0825	0.27651	0.02808			3.712
Flushing toilet	Male	147	0.8163	0.38854	0.03205	3.964	0.048	1.01
	Female	97	0.7629	0.42752	0.04341			0.99
Swimming pools	Male	147	0.2585	0.43931	0.03623	54.531	<0.001	3.269
	Female	97	0.0928	0.29164	0.02961			3.541
Drinking	Male	147	0.1769	0.38286	0.03158	41.476	<0.001	2.915
	Female	97	0.0515	0.22226	0.02257			3.229
Generating Electricity	Male	147	0.7483	0.43547	0.03592	1.636	0.202	-0.63
	Female	97	0.7835	0.41399	0.04203			-0.637
Construction of Buildings	Male	147	0.7415	0.43931	0.03623	0.459	0.499	0.342
	Female	97	0.7216	0.45052	0.04574			0.34

From the initial hypothesis, the findings revealed that (i) the hypothesis “education will significantly influence the degree of acceptability of the proposed reuse projects in the Canaanland region” is accepted as findings revealed a similarity in the decisions of the respondents on treated WWR for several applications (ii) the hypothesis of “knowledge of the community on global water shortages will be significantly higher” was rejected given that 64% of the respondents had little knowledge or no idea at all on global water shortages. (iii) the hypothesis on “non-contact reuse projects will be mostly accepted in the Canaanland community” can be accepted as findings indicated the willingness of the respondents to accept treated WWR projects that involved little or no contact.

## 4 Discussions

According to Adewumi et al. (2014) the resistance from potential customers has been the primary reason for the failure of most water schemes that utilize reclaimed wastewater. Findings revealed that a majority of the respondents understood the economic and environmental importance of recycling and reusing treated wastewater but were concerned with the execution of the reuse scheme. For instance, a majority of the respondents agreed that wastewater reuse would be beneficial as fertilizer alternatives in agricultural production; this would help reduce the number of pollutants in the environment via indiscriminate wastewater discharge. Also, most of the respondents agreed to the financial benefits of wastewater reuse. Additionally, some respondents acknowledged the job creation potential of wastewater reuse schemes, especially in developing countries in a qualitative assessment. Findings show that about 108 respondents believed that the current water pumping rate within the university campus was unsustainable. However, the respondents identified some concerns that could affect their acceptance of a wastewater reuse scheme, with the most significant concerns being the quality of the recycled water, health reasons, poor plant management, and lack of trust for the treatment process.

Furthermore, the respondents were most likely to accept treated WWR schemes as long as the degree of human contact or consumption is minimal. The most preferred reuse projects by the respondents were flushing of toilets, construction of buildings, electricity generation, washing cars, which had over 70% acceptance by the respondents. The study revealed that people were less likely to accept treated WWR for potable purposes. The respondents were also willing to accept a treated WWR project as long as it receives endorsements from medical doctors, professors, and experts in that field and state and federal government.

In comparison with previous studies, there were certain similarities regarding the most preferred reuse schemes. For example, Baawain et al. (2020), Fielding et al. (2019), Massoud et al. (2018), Mu'azu et al. (2020), Redman et al. (2019), and Smith et al. (2018) all reported that users were aware of the economic and environmental importance of reusing treated wastewater and were willing to accept reuse projects as long as it involves fewer contacts with the users. Customers preferred reuse schemes such as watering of lawns, flushing of toilets, car wash, and fertilizer for non-edible crops according to research. There were, however, some peculiarities in some studies that prompted the decisions on the respondents. For example, customers of respondents in the middle east were most likely to accept reuse schemes as a result of the high cost of desalination of seawater to augment freshwater supplies and thus were willing to pay a fee as wastewater maintenance or recycling tax (Abdelrahman et al., 2019; Baawain et al., 2020; Mu'azu et al., 2020).

Also, a survey carried out by (Robinson et al., 2005) shows that both men and women are not comfortable with wastewater reused for potable purposes but consider treated WWR as highly acceptable in the areas of firefighting, agricultural uses, lawn watering, and car wash. In this study, people with at least a university qualification saw treated WWR as the right course as compared to the citizens without a university education. This study was similar to a United States research, which shows that people are positive with wastewater reuse so long it is a low-contact process. In this study, 96% of the people accept wastewater for park irrigation, 95% accept treated wastewater to be utilized in sidewalk landscaping, and 94% do not mind its applications in the industrial sector (Friedler and Lahav, 2006). Another study carried out in the United States reveals that there would be a higher treated WWR acceptance given that the protection of the environment is a definite advantage of the treated wastewater reuse scheme, and the role of the treated wastewater to be reused is clearly stated (Hartley, 2006). This is similar to (Smith et al., 2018), which suggests that the reuse of treated wastewater would be highly accepted as long as it tackles environmental issues, water conservation issues, and a high level of public awareness is available. Also, on that line (Prajapati, 2018) suggests that the citizens of Copenhagen, despite the demographic differences, widely accepted the concept of treated WWR so long the household supply facilities have separate treatment facilities (Prajapati, 2018).

A study carried out by (Wilson and Pfaff, 2008) in Durban, South Africa, also shows people's unwillingness to accept treated wastewater as a source of potable water supply but suggests that big industries reuse treated wastewater before households. For irrigation, a study was carried out by (Carr et al., 2011) in Jordan on farmers who already utilize treated and raw wastewater for irrigation purposes. Most of the identified farmers viewed the use of treated wastewater for irrigation positively and recognized its nutrients potentials. The study revealed that the farmers who had a favorable view of treated WWR for agricultural purposes had facilities to manage their wastewater one way or the other (Carr et al., 2011).

A study carried out in Nevada revealed that suburban dwellers supported the reuse of reclaimed water as compared to their urban and rural counterparts for irrigation, lawn watering, and others (Redman et al., 2019). In another study, farmers were more receptive to treated WWR than the general public (Ricart et al., 2019). This study, however, suggests product certification, recycled water consortiums, and regular visits of public to treatment sites as methods to foster understanding amongst the general public on the concept of treated WWR (Ricart et al., 2019). A similar study carried out by (Khanpae et al., 2020) shows that the psychological support of farmers could go a long way in the effective implementation of treated WWR as farmers adopted treated wastewater as an economically and socially acceptable technology. In Oman, studies show that the communities were very likely to accept treated wastewater for the irrigation of non-edible crops, groundwater recharge, and industrial activities (Baawain et al., 2020).

Motivators such as cost, ecological conservation, current, and future water shortages were identified as triggers to treated WWR acceptability by people according to a study carried out by (Rice et al., 2019). A study, on the other hand, carried out by (Massoud et al., 2018) revealed that religious beliefs go a long way in affecting the acceptability of treated WWR. This study showed that the reuse of wastewater for any purpose at all was two times more likely to be rejected by very religious people as they believed that wastewater reuse contradicts their beliefs (Massoud et al., 2018). However, religion had a negligible impact on the public perception of reusing treated wastewater in Canaanland. Age, monthly and annual incomes, level of education, and gender were the factors that affect the acceptability of wastewater reuse by individuals, as identified in a survey carried out by (Zabala et al., 2019).

Furthermore, based on the general awareness of the public on global water shortages, knowledge on wastewater reclamation, treatment, and reuse, the findings revealed a general knowledge gap on these issues. Given that 45.5% of the respondents were students, the lack of awareness on the subject indicates the failure of the relevant authorities to pass on information on the state of water in the various parts of the country. Also, the lack of data in most parts of Nigeria poses a challenge and could affect the knowledge of the respondents on the topic of water shortages. Furthermore, the continuous availability of water within the Canaanland community goes a long way in affecting the community's view on recycling and reusing treated wastewater. The residents were unaware of the water situation in other parts of the country and the world at large. Besides, 17% of the respondents only realized the importance of treated WWR only when they were directly affected by water shortages. Similar findings were made in the United Arab Emirates (Abdelrahman et al., 2019). In their study, 70% of the respondents were unaware of water shortage challenges in the region. This was also similar to a study by Baawain et al. (2020) in Muscat, where only 32.4% of the respondents were aware of wastewater and wastewater treatment. However, the gap between the people who were unaware of the water shortage situation in the country and the people who were aware was not too wide when compared to studies highlighted earlier. This may be attributable to the fact that the Canaanland study comprised of a high number of respondents from the academic environment. Also, the cost implications of groundwater abstractions and the unsustainability of groundwater abstraction to meet all the needs within the Canaanland community raised some concerns among the respondents. The need for information dissemination by the city authorities on treated WWR is necessary to sensitize the residents on treated WWR.

In the area of endorsements, cities with large farms and the semi-urban regions were more likely to accept treated WWR as a fertilizer replacement in agricultural production. Thus, they would prefer the endorsements of local farmers and local municipalities (Carr et al., 2011; Redman et al., 2019; Ricart et al., 2019). In Canaanland, the results revealed that customers cared less about endorsements from farmers and industrialists but rather preferred endorsements from medical doctors and university professors and experts. Understanding the public perception of the concept of wastewater reuse will enable developers to identify possible areas in which wastewater can be optimally reused without causing many controversies. Table 9 gives a summary of more perception studies and their outcomes, as extracted from literature.

**Table 9:** Summary of Some findings from literature.

Source	Location	Most Preferred WWRS	Unaccepted WWRS	Major Concerns	Findings	Recommendation
Robinson et al. (2005)	USA	Firefighting; car washing; lawn irrigation; agricultural usage	Potable Reuse; Supplementing Groundwater; Laundry		People with lower income, less education, and people over 65 had little knowledge of WWR	Making information readily available
Friedler et al. (2006)	Israel	Sidewalk landscaping; WC flushing; firefighting.	Domestic laundry; Food preparation, potable aquifer recharge	Health concerns	Water saving, savings in infrastructural cost, and environmental improvements were considered to be reasons to support WWR.	Proper treatment and endorsements from experts
Friedler and Lahav (2006)	Israel	Irrigation of parks; sidewalk landscaping; use in the construction industry	Commercial launderettes	Health concerns	Low contact reuse projects were more likely to be accepted.	National authorities are advised to set up public campaigns, discuss health-related concerns, highlight the economic opportunities of WWR
Buyukkamaci and Alkan (2013)	Turkey	Toilet Flushing; Road Washing; Construction; Firefighting; Agricultural Irrigation; Industry Use	Potable Reuse	Public Health Risks	Reuse scheme not involving close human contact were most likely to be accepted	Active policy initiatives and public awareness
Wilson and Pfaff (2008)	South Africa	N/A	Potable Water Reuse	Emotional Concerns (Yuk Factor); Technical competency; Environmental concerns	Religion had no direct impact on WWR; Respondents suggests that the direct beneficiaries must bear project costs; Unplanned reuse was more favored than planned reuse	Wastewater management case for potable reuse may entail a distinct methodology than arguments with immediate water shortage drivers
Adewumi et al. (2010)	South Africa	Landscape Irrigation; Industrial Processes	Potable Water Reuse	Public Health; Tariffs	Parameters such as aridity, tariffs, retrofitting and new installations, guidelines, and reuse regulations, public health, the quantity of reuse were most likely going to affect the decision of the public	The parameters stated in the findings section must be addressed
Maleksaeidi et al. (2018)	Iran	Agricultural activities	N/A	Health concerns; social impacts; environmental impacts	The study explained that the most critical drivers for employing untreated wastewater for irrigation by farmers were water scarcity, increasing crop yield, difficulty to access freshwater, saving freshwater, increasing soil fertility, and decreasing production costs.	The study emphasized the necessity for planning to improve wastewater treatment along with suitable policies and procedures to enhance farmers' commitment to environmental conservation and human health.
Baawain et al. (2020)	Oman	Irrigation of non-edible crops; landscape irrigation; firefighting; cool buildings.	Potable Reuse; Discharge to the marine environment	Health Concerns	outcomes suggested that the residents were optimistic about supporting any possible option that favored human health and the environment	Public concerns will need to be addressed using research outcomes.

∗WWRS: Wastewater Reuse Scheme; ∗WWR: Wastewater Reuse.

## 5 Opportunities and implications for developing countries

### 5.1 Opportunities

Managing wastewater resources should be considered as a critical role in IWRM. Ignoring wastewater could lead to adverse environmental pollution, which could deteriorate public health and the ecosystem at large. Also, wastewater, if professionally managed, could be economically beneficial to any given society (Garcia and Pargament, 2015; Hagenvoort et al., 2019; Omole et al., 2019).

Many developed nations have created a sustainable framework and technologies to treated WWR for several essential purposes and even for potable reuse. These schemes capitalize on advanced techniques for wastewater reclamation and reuse and thus may turn out to be highly expensive processes (Bailey et al., 2018; Ghernaout and Elboughdiri, 2019; Giagnorio et al., 2019; Marron et al., 2019; Wei et al., 2020; Wei et al., 2020). Advancements of treated wastewater reuse schemes have slacked in many developing countries, primarily due to the economic conditions when compared to their developed counterparts. Furthermore, the treatment and development of some of these wastewater treatments and reuse technologies from several findings may not be widely accepted for some purposes (e.g., potable reuse) in many developing countries due to several challenges. These challenges could range from economic instability, political instability, or outright disapproval from the local communities. The maintenance and operation of wastewater treatment facilities for reuse projects might pose to be too expensive for a lot of modern or rural communities in developing nations.

Many developing countries may have a challenge of establishing complex reuse schemes and policies, given the financial constraints, amongst other factors (UN-HABITAT, 2014). However, opportunities present themselves for developing countries with peculiar situations, especially in the areas of agriculture. For example, the USA rarely adopts reclaimed or treated wastewater for irrigation purposes, even with its advanced regulatory standards governing the reuse of treated wastewater for irrigation. This is not the case with developing countries, predominantly arid regions that frequently employ partially treated or raw wastewater for crop cultivation (Gerba and Choi, 2009). Direct reuse of wastewater has proven to be detrimental to public health, soil salinization and, groundwater pollution; therefore, the need for cheap or proper treatment processes will be required for developing countries (Navarro et al., 2015; Shakir et al., 2017). For developing communities, where wastewater is not suitable for direct applications, decentralized treatment techniques could be deployed to reduce the contamination levels of the water before use. Urban communities can lower the cost of wastewater treatment by creating suitable decentralized treatment systems based on their financial capacities and source of funding, thus making for an economic reuse prospect (SSWM, 2020). The possibilities for adopting decentralized reuse are presented below:

i. Waste Stabilization Ponds
ii. Constructed Wetlands
iii. Aerated Ponds
iv. Non-Planted Filters
v. Anaerobic technologies such as biogas settlers, anaerobic digestion, and anaerobic baffled reactors.
vi. High tech options such as membrane reactors, rotating biological contactors, activated sludge, advanced oxidation processes, ozonation, and anammox.

These decentralized solutions for treatment and reuse purposes would allow individual communities in developing countries to harness new possibilities in wastewater reclamation and reuse based on the acceptance within those communities. With a properly developed decentralized approach, developing communities may not have to rely on public or federal governments to develop overly expensive centralized systems. Sub-Saharan African Nations, which suffer from inadequate city planning, could benefit from a decentralized system for wastewater management, thus tackling the specific unique needs of those individual communities. In northern Nigeria, for example, where agricultural activities are common, decentralized systems could be beneficial to aid local farmers tackle the constant threats of drouths and ever-decreasing crop and livestock yields (Chianu et al., 2004). However, every community might have other pressing needs or concerns which could influence the decision of the local communities.

From the findings of this research, several opportunities that align with developing sustainable solutions to the customers or respondent's preference regarding treated WWR will present themselves. Sustainable solutions to treated WWR have been identified by several scholars that could be emulated in developing regions in the areas of flushing toilets, building construction, electricity generation, car wash, watering lawns, firefighting, etc. These were the most preferred reuse schemes by respondents. Table 10 gives an insight into some sustainable solutions and technologies developed by scholars to aid the reuse of wastewater resources efficiently.

**Table 10:** Approach/Technologies to the most accepted WWR reuse projects from literature.

Source	Problem Tackled	Approach/Technologies	Findings	Recommendation
Ren et al. (2019)	Flushing Toilet	Using a membrane bioreactor and a biological aeration filter, Graywater suitability for flushing of toilets was determined.	The Gray water treated using the membrane bioreactor contained no bacteria after being treated for 15 days	Graywater, purified by a membrane bioreactor, should be adopted for flushing of toilets as it was highly viable.
Taemthong (2018)	Flushing Toilet	Greywater from washbasins was passed through three distinct methods of treatment. The treatments adopted include a sedimentation tank, sand and carbon filtration system, and a 24-hour aeration tank.	i. No fecal coliform and E. Coli were found in the treated wastewater. ii. TSS, BOD_5,_ and Turbidity were reduced to an efficiency of 93%, 75%, and 91%, respectively. iii. 69% of toilet users indicated that the treated greywater was similar to tap water	Treatment of greywater from washbasins with simple sand filtration techniques, for instance, proved to efficient
Bose et al. (2018)	Electricity Generation	Using a 2-chambered microbial fuel cell, fabricated with carbon cloth electrodes and Nafion-117 membrane with a platinum catalyst, the energy was recovered while treating wastewater.	i. The production of 810 ± 10 mW/m^2^ of power was achieved using microbial fuel cell ii. Microbial fuel cells were seen to be effective in cleaning wastewater and generating Electricity simultaneously	Bacteria present in wastewater are effective in electricity generation while breaking down wastewater due to their electroactive nature. Further investigations need to be carried out on this to see how this can be harnessed for large scale applications.
Kawale et al. (2017)	Electricity Generation	Adopted a two-chambered Microbial fuel cell inoculated with a mixed culture of cellulose-degrading bacteria to generate Electricity.	A power density of 469.48 W/m^2^ was achieved with a maximum voltage of 1.0 V.	It is recommended that the constituents of wastewater be determined to generate power from it optimally.
Duke et al. (2020)	Firefighting	The study utilized a process of flocculation, ozone, powdered activated carbon, and ceramic membrane filtration to treat wastewater meant for firefighting.	The powdered activated carbon aided in reducing the rate of fouling of the membrane when compared to operating the membrane without the carbon dosing.	It is feasible to treat wastewater intended for firefighting without compromising on standards.
Rodriguez Boluarte et al. (2016)	Car washing	Treatment processes such as ozonation, membrane bioreactor, and coagulation were deployed to treat wastewater from carwash for reuse.	This study showed that the use of ozonation was more effective than coagulation processes in the removal of suspended solids and chemicals. Membrane Bioreactor, however, showed a greater potential of removing 100%, 99%, 97.3, and 41% of suspended solids, COD, TOC, and ammonia, respectively.	Membrane bioreactor is seen as having great potentials in the recycling of wastewater from carwash for reuse.
Uçar (2018)	Car washing	Treatment processes such as settling, filtration, and membrane filtration were adopted in the study	i. The settling process reduced the number of suspended solids in the wastewater. ii. The removal of COD was negligible when adopting filtration techniques. iii. Membrane filtration aided in the removal of 60–76% of COD.	Car wash effluents could be treated using settling and membrane filtration.

From Table 10, different projects were picked based on their wide acceptance in the study location, and solutions from several scholars were summarized to see their potentials in developing countries. For example, several treatment processes have been seen to be useful for optimizing wastewater resources in any given community. For instance, treated wastewater from washbasins was comparable to tap water by users in a study by (Taemthong, 2018). This implies that with the right endorsements from experts as expressed in this study, users and potential customers in urban communities may be willing to adopt treated wastewater for flushing toilets as they have little to no contact with the water. The method adopted by (Taemthong, 2018) revealed some cost-effective wastewater treatment methods such as sedimentation tanks, to reduce the number of suspended particles in washbasins. Harnessing wastewater from washbasins in newly developed cities within developing countries could be possible as it could be integrated into city planning. In Nigeria, upcoming urban development schemes such as Greater Port Harcourt city (Rivers state), Eko Atlantic (Lagos State), Centenary city in the Federal Capital Territory (FCT), and Abuja (FCT) could benefit from WWR integrated into their development.

The treatment of wastewater for the generation of sufficient Electricity could be sustainable, especially for developing cities in third world countries which suffer from the inadequate power supply. Studies have revealed that the adaptation of cost-effective microbial fuel cells for energy recovery in wastewater treatment could serve as a sustainable energy solution (Naik and Jujjavarappu, 2020; Tatinclaux et al., 2018). From their study, the future of energy recovery from treated wastewater will be microbial fuel cells. Naik and Jujjavarappu (2020) further revealed that wastewater from sugar factories has higher potentials for energy recovery based on their physicochemical properties, which are suitable for better power output.

Furthermore, from an economic standpoint, urban communities in developing countries could benefit immensely from cheap wastewater treatment and reuse schemes. Omole et al. (2019) revealed that Canaanland, Nigeria, which is the pivotal point of this research, could make approximately $107,000 per annum and save $38,000 quarterly on energy expenditure when treated wastewater is reused using a well-maintained constructed wetland. However, this economic evaluation was carried out considering only a few projects and thus may vary significantly when the widely accepted projects are taken into consideration. Also, sustainable surface and groundwater withdrawal and corporate social responsibility (CSR) were reported to be added benefits of a proper wastewater reuse scheme (Jeremy, 2015; Omole et al., 2019).

Some cities in developing countries have taken their wastewater reuse potentials to another level. Presented in Table 11 are eight cities from developing countries that have pioneered wastewater development for a while and have aligned themselves to attaining SDG number 6. The eight cities presented in Table 11 are in nations with the same economic characteristics as Nigeria, where the study was performed, hence their selection. All the selected nations are middle-income economies except for Uganda, which is a low-income economy (World Bank, 2019). Therefore, nations in a similar economic bracket could take a clue from the achievements of these nations and develop a similar reuse scheme tailored to their urban and national needs and acceptability. The areas highlighted in Table 11 are the volumes of wastewater generated by the individual cities, the volume of wastewater treated by the cities, and the volume of wastewater reused. The purpose of reuse has also been identified, considering fertilizer and energy recovery. Some of the cities have shown high potentials in the reduction of Green House Gas emissions and have created a roadmap for their city's future.

**Table 11:** Cities from developing countries with a roadmap for WWR. Source: IWA (2018).

S/N		1	2	3	4	5	6	7	8
Developing City		Aqaba	Bangkok	Beijing	Chennai	Durban	Kampala	Lima	Manila
Country		Jordan	Thailand	China	India	South Africa	Uganda	Peru	Philippines
Population (2016)		194,000	5.6 million	21.7 million	8.5 million	3.7 million	1.5 million	10 million	12.2 million
Projected Population (2030)		258,000	7 million	23 million	11 million	4 million	4.5 million	12 million	13.5 million
Wastewater Generated	Sewer Service Coverage	90%	40%	95%	100%	16%	40%	83%	15%
	On Site Sanitation	10%	60%	5%	0%	84%	60%	17%	85%
Treated Wastewater		100% = 45 ml/d	100% = 1.3B l/d	88% = 4.48B l/d	70% = 769 ml/d	100% = 108 m l/d	100% = 87 ml/d	15% = 240 ml/d	100% = 510 ml/d
Treated Wastewater Currently Reused		69%	5%	15%	49%	44%	100%	5%	0%
Citywide GHG Emissions	ton CO_2_/year	N/A	N/A	173m	3.82m	27.1m	N/A	15.4m	29 m
Potential to Reduce Emission	ton CO_2_/year	-81,000	-638,000	-1044000	-235,000	-438,000	-114,000	-652,000	-168,000,000
Energy Recovered		100%	62%	45%	77%	8%	227,000 KWh/year	low	low
Fertilizer Recovered		No	Yes	Yes	No	Yes	Yes	No	Yes
Notable Achievements and roadmap		4 million USD income generated; set to reuse treated wastewater in hotels and tourism activities by 2035	Sludge collection is creating new business opportunities; fertilizer plants from wastewater set to be increased by 2030	47%, 30%, and 20% of wastewater reused for irrigation, environmental reuse and industrial reuse respectively; plans to expand infrastructure to distribute wastewater to the community by 2030	15% of the city's water demand met via recycling; to achieve 100% recycling by 2030	There has been a reduction in effluent being discharged into the environment by 10%; set to use 96 mgd for potable purposes by 2030	Biogas recovery has reduced GHG emissions significantly; set to totally rely on biogas for power	about 3400 hectares of land irrigated with recycled water; set to develop its wastewater reuse infrastructure by 2035	The framework set up by the legislative arm to commit stakeholders to 100% coverage and safely managed/reuse of Wastewater and sludge by 2028.

In summary, Table 11 showcases eight (8) cities that have established a roadmap to the establishment and development of wastewater reuse facilities to increase economic stability within the cities. Kampala, for example, applies 100% of its treated wastewater resource for the generation of electricity, and they are set to continue increasing their infrastructure up to the year 2030. Aquaba, a city in Jordan, has implemented a zero-discharge policy to protect their marine environment, which the city relies on for tourism. It achieves this by adopting a decentralized approach. Wastewater has served highly functional in Aquaba in the areas of the greening of the urban landscape and reducing carbon emissions. Aquaba, Bangkok, Durban, Kampala, and Manila are known to treat 100% of its wastewater for reuse, with Durban planning a wastewater reuse scheme for potable purposes. This is a far cry from the sub-Saharan African nations where most of the wastewater generated is left untreated, thus becoming a source of pollution, thereby triggering waterborne diseases such as cholera and diarrhea (United Nations University, 2019). Cities such as Kumasi, Nairobi, Dakar, and Bulawayo in Ghana, Kenya, Senegal, and Zimbabwe are reported to utilize untreated wastewater for agricultural purposes. The problem with most of sub-Saharan Africa is the unavailability of data (United Nations University, 2019).

#### 5.1.1 Potential government policies and framework

**Figure 7:**
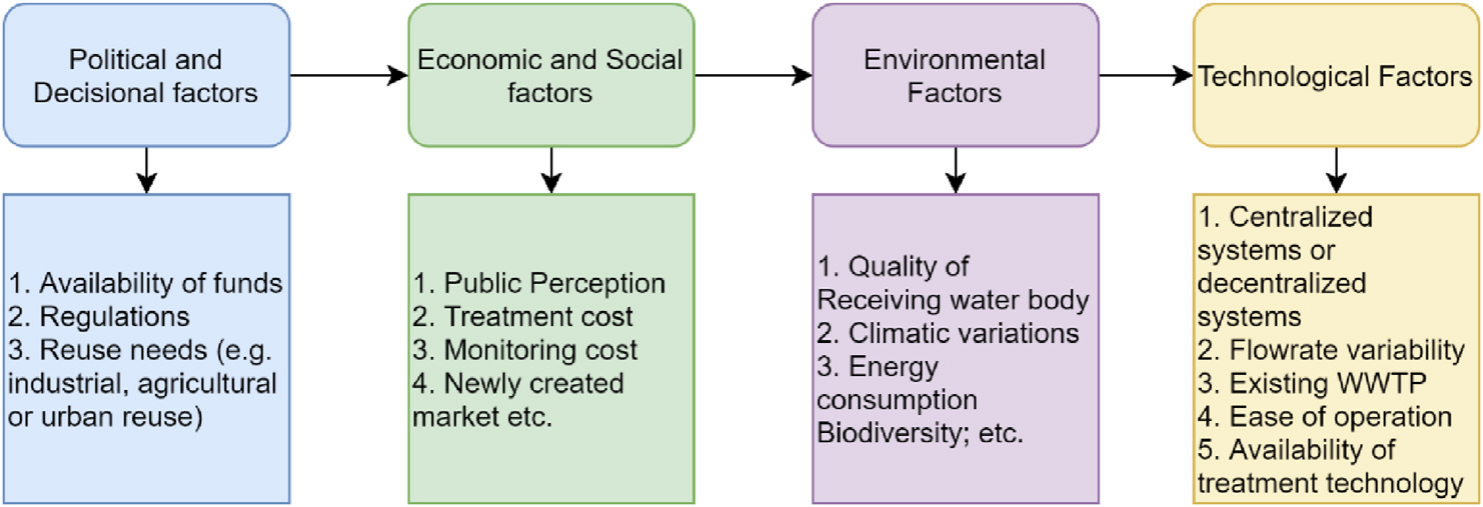
Holistic approach to wastewater reuse. (Source: Sgroi et al., 2018).

For a possible adaptation of treated WWR schemes in other developing countries, policymakers and the regulatory agencies need to understand the complexities and interlinkages of wastewater and water projects before venturing into treated WWR projects (Schwabe et al., 2020). Sgroi et al. (2018) developed a holistic approach that should be followed critically in treated WWR development. The study highlighted several factors that could influence treated WWR projects. These factors include political and decisional factors, economic and social factors, environmental factors, and technological factors, as summarized in Figure 7.

In the case of Nigeria, the National Environmental Standards and Regulations Enforcement Agency (NESREA), is responsible for overseeing the sustainable protection and development of the Nigerian environment, conservation of national biodiversity and also, the development of the country's Natural resources (Mantu, 2019; NESREA, 2020). The commission has developed a guideline for effluent discharge standards that industries, institutions, and other wastewater producing sectors must comply with to avoid possible sanctions. However, the NESREA (Establishment) Act of 2007 is vague about the possibilities of reusing treated wastewater for several purposes (NESREA, 2009). The Act only concentrates on effluent discharge rather than reuse. Also, several drawbacks such as poor data management, poor enforcement of existing laws, weak institutions, insufficient funding of environmental protection schemes, overlapping objectives with the Ministry of Water Resources persists (Ladan, 2013; Nwankwoala, 2011; Omole, 2013; Omole et al., 2019).

Given the drawbacks in the NESREA act, the Nigerian government would have to consider several options to cater for these loopholes:

i. A Public-Private partnership needs to be established between the government and private cities or communities. In this case, the treatment and reuse of reclaimed wastewater will be carried out by those individual communities, so long as they comply with the national effluent discharge standards. This will also bring out the uniqueness of each city, given that the public perception of communities on treated WWR in the country may differ from city to city.
ii. The monitoring of treated WWR projects should be done at the community level. Locals should be trained to make sure a routine monitoring and quality assurance is guaranteed at all times. This technique will also help to create jobs for citizens and ease the burdens on the national agency. However, frequent reports will have to be submitted by the monitoring team of the treated WWR projects and the municipal representative.
iii. Given the low levels of awareness on the subject of treated WWR, sensitizations and education need to be promoted at the community level. Also, ads and campaigns should use the internet and social media sources to communicate environmental issues, given that it was the primary source of information identified by the respondents in this study.
iv. A frequent review of existing treatment, effluent discharge, and reuse guidelines must be performed to keep communities safe at all times (Kayode et al., 2018).
v. Overlapping objectives must be solved by merging two similar agencies in a nation. Also, an emphasis must be placed on economic returns, thus creating a lucrative market for treated WWR (Akpan and Olukanni, 2020).

**Figure 8:**
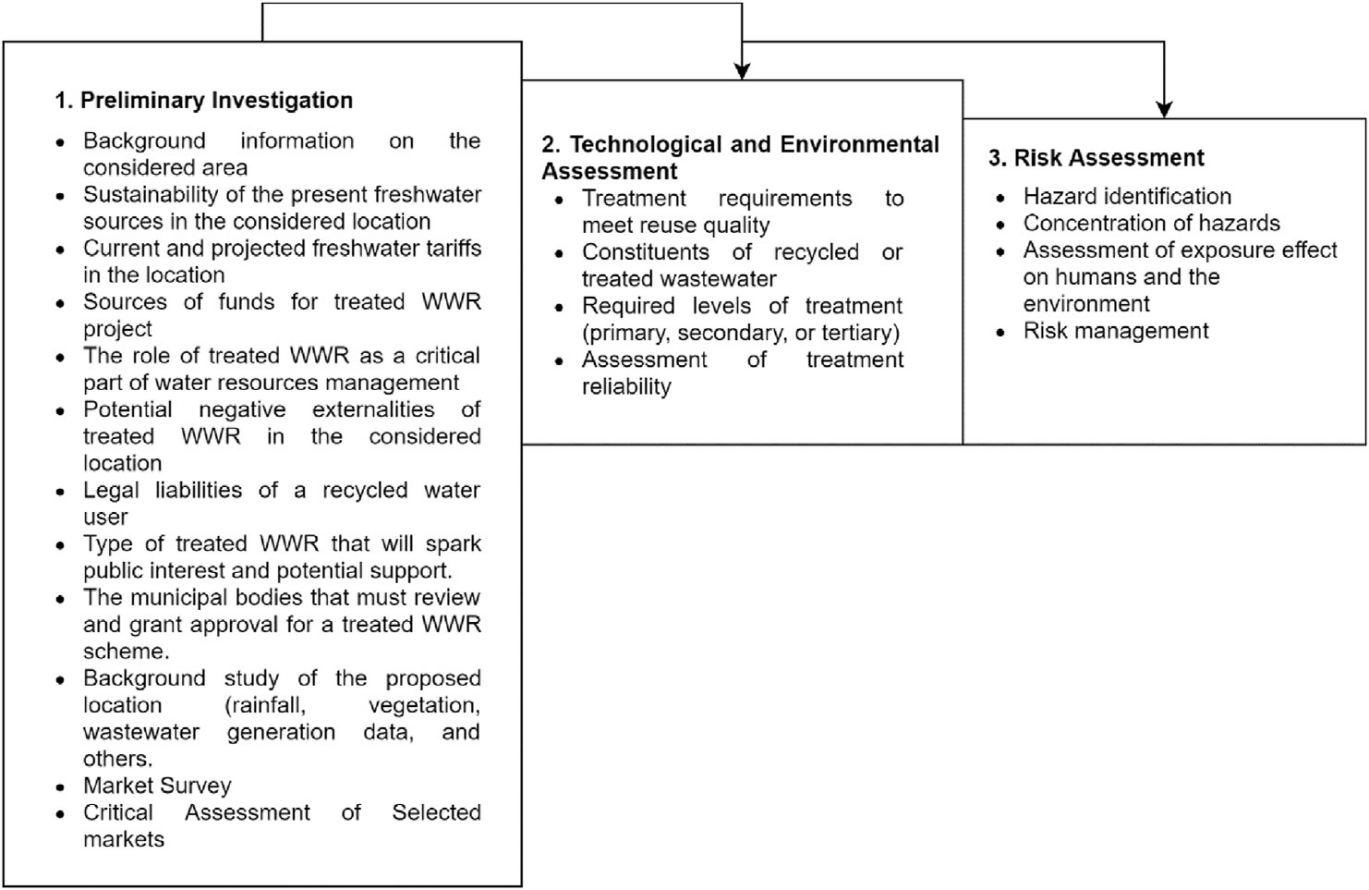
Requirements to consider when setting up a WWR scheme in developing communities.

In order to hand over the planning and execution of treated WWR schemes to private and municipal authorities, NESREA will have to ensure that some critical requirements are met by the executing body using careful evaluation procedures. A 3-phase approach was developed for potential planners of treated WWR programs to follow to attain success. This 3-phase approach was inspired by a wastewater planning study carried out by Adewumi (2016). Figure 8 gives a summary of the requirements that must be assessed before commencing treated WWR schemes. The 3-phase approach involves a preliminary investigation, technological and environmental assessment, and risk assessment.

### 5.2 Implications

Decentralization shows up as a coherent answer to tackle maintainability issues of wastewater challenges in developing counties frameworks, as it centers around the on-location treatment of wastewater (Capodaglio, 2017). Given the advancement of treated WWR projects in some developing countries, some communities have outgrown their treatment facilities and could thus require an upgrade or a complete overhaul of existing treated WWR infrastructure. The Canaanland community's wastewater infrastructure, for instance, has not developed simultaneously with the rapid infrastructural development taking place. The city operates a fairly maintained constructed wetland, which will not be sufficient for a sustainable WWR scheme. A report by (Isiorho and Oginni, 2014) indicates that the storage tanks within the WWTP have an estimated volume of 1,054,000 L. This implies that the wastewater volumes (1.5 million liters) reported by (Isiorho et al., 2014; Omole et al., 2017, 2019) have exceeded the treatment plants' capacity. Furthermore, studies aimed at ascertaining the quality of the discharged effluents have revealed a high concentration of phosphate, lead, and iron (Adewumi et al., 2009; Omole et al., 2017). These effluent discharge to a nearby river has significantly affected the local communities who depend on that river for their daily activities (Omole et al., 2019). For Canaanland and similar emerging cities to maintain an acceptable effluent quality, the treatment facility will have to be entirely remodified or redesigned (Adewumi and Ogbiye, 2009; Isiorho et al., 2014).

**Figure 9:**
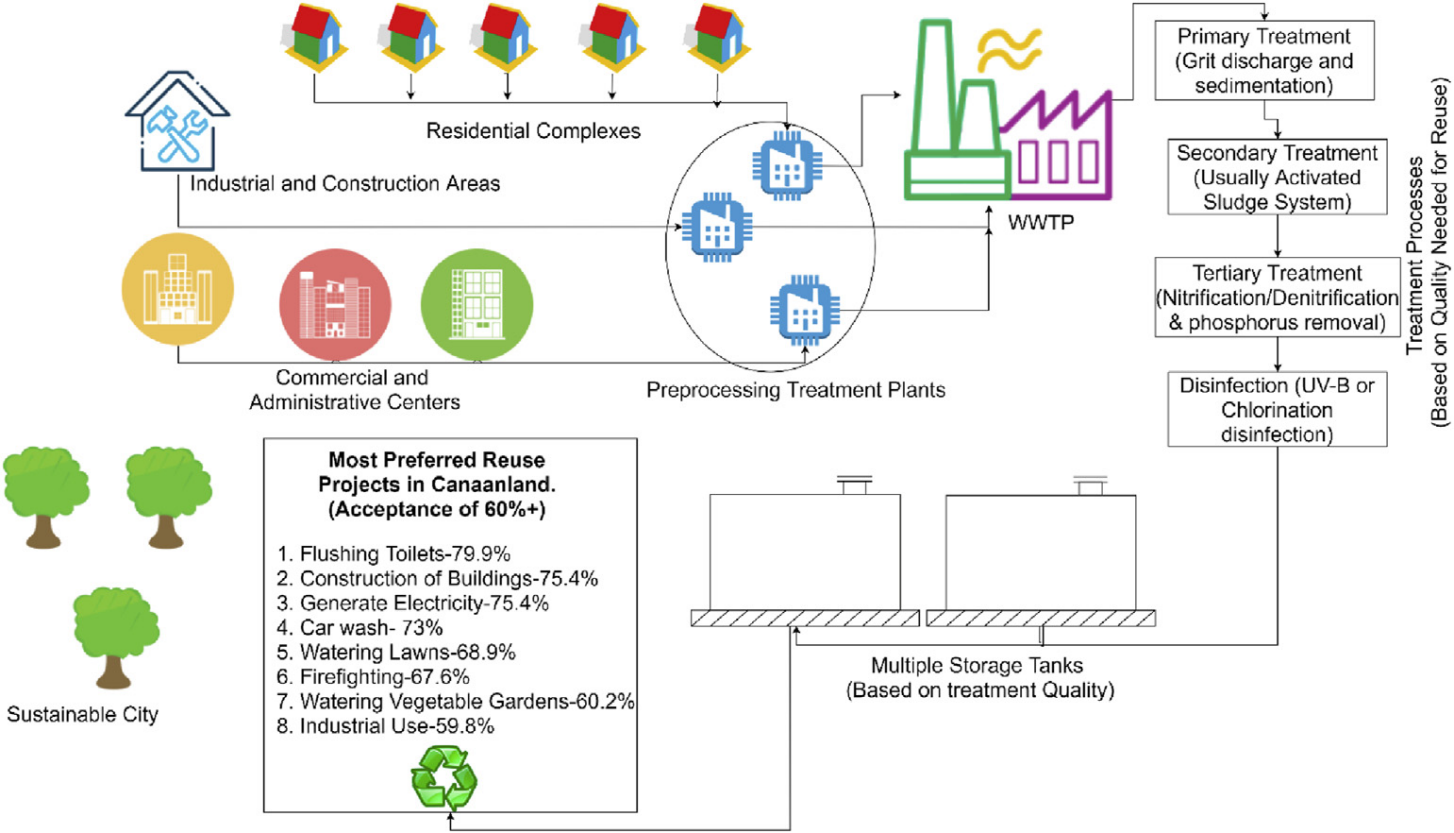
Proposed flow process for treated WWR for developing cities.

Developing a sustainable WWR scheme in urban communities in developing countries might require a centralized or decentralized approach to wastewater management, depending on the peculiarities and needs within those cities. This study has been able to create a simple flow process for WWR that could aid secure the future of some cities. This process flow is represented in Figure 9. From Figure 9, wastewater should be separated at source and channeled through different pipelines to some preprocessing plants that have been designed to process wastewater based on its constituents. For instance, wastewater from residential areas will possess different characteristics when compared to wastewater from administrative buildings. Also, the preprocessing treatment plants could be used for wastewater stream segregation, to reduce the burden of high volumes reaching the centralized treatment plant. In this case, preprocessed wastewater can be sent back to the city area for reuse. The preprocessed wastewater with potentially hazardous substances that could not be removed or sorted in the preprocessing plants can be discharged into the main wastewater treatment plant, where it undergoes different degrees of treatment based on the desired outcome. Stream segregation has been reported to be beneficial in the areas of cost reduction and ease of final treatment (Beler-Baykal, 2015; Doǧruel et al., 2003; Khan et al., 2011). This treated wastewater can be stored in adequately maintained storage tanks and pumped to the necessary reuse points. For developing cities, the reuse should center mainly around the most widely accepted reuse projects obtained from this study.

## 6 Conclusion

The success of treated WWR is based on its ability to integrate practical and strategic planning. This study has given an insight into the public's perception of treated WWR within the city, thus allowing decision-makers to understand why its residents may or may not accept treated WWR. The study has revealed that the residents appreciate the economic and environmental importance of treated WWR but will require reuse projects that would involve little human contact. However, integrating professional endorsements from medical doctors, professors, and experts could help convince the residents to embrace treated WWR. Furthermore, an advanced decentralized or centralized approach, merged with cost-effective and efficient treatment avenues for wastewater management could ease the development of a treated WWR scheme. Findings from this study can serve as a platform for decision making and hypothesis for future research in newly developed or developing cities in Nigeria and on university campuses, given the large percentage of respondents who were students. However, some areas within Nigeria and across the world may have different perspectives. For instance, religious views played an insignificant role in treated WWR acceptability. This may not be the case in other cities across the world. Also, the lack of awareness on water shortages within the Canaanland community shows that people need to be informed about what is going on in other parts of the globe, and sensitizations need to be passed across to the general public on methods of mitigating water scarcity challenges in the region. Studies will need to be conducted in other parts of the country to determine their peculiar situations and understand the best means to communicate necessary environmental information across. Due to the Covid-19 pandemic and the imposed lockdowns, extensive surveys were hindered. However, for future studies in the Canaanland region, a larger sample size should be assessed to include surrounding communities that may have a different economic and educational background. Understanding the opportunities and implications of treated WWR in developing or emerging cities will align them on the right path of achieving the SDG 6 at local levels and, thus, create a sustainable future for the environment and humans at large.

## Declarations

### Author contribution statement

Victor E. Akpan: Conceived and designed the experiments; Performed the experiments; Analyzed and interpreted the data; Wrote the paper.

David O. Omole: Conceived and designed the experiments; Wrote the paper.

Daniel E. Bassey: Analyzed and interpreted the data; Contributed reagents, materials, analysis tools or data; Wrote the paper.

### Funding statement

This research did not receive any specific grant from funding agencies in the public, commercial, or not-for-profit sectors.

### Competing interest statement

The authors declare no conflict of interest.

### Additional information

The following are the Supplementary data related to this article:

## References

[bib1] Abdelrahman R., Khamis S., Rizk Z. (2019). Public attitude toward expanding the reuse of treated wastewater in the United Arab Emirates. Environ. Dev. Sustain..

[bib2] Abu-Madi M., Al-Sa’ed R., Braadbaart O., Alaerts G. (2008). Perceptions of farmers and public towards irrigation with reclaimed wastewater in Jordan and Tunisia. Arab Water Council J..

[bib3] Abu-Madi M., Mimi Z., Abu-Rmeileh N. (2008). Public Perceptions and Knowledge towards Wastewater Reuse in Agriculture in Deir Debwan. https://fada.birzeit.edu/handle/20.500.11889/4261.

[bib4] Abubakar I.R. (2019). Factors influencing household access to drinking water in Nigeria. Util. Pol..

[bib5] Adewumi I., Ogbiye A.S. (2009). Using water hyacinth (Eichhornia crassipes) to treat wastewater of a residential institution. Toxicol. Environ. Chem..

[bib6] Adewumi J.R. (2016). Planning of wastewater reuse programme in Nigeria. J. Sustain. Dev..

[bib7] Adewumi J.R., Ilemobade A.A., Van Zyl J.E. (2009). Planning model for wastewater reuse system in South Africa. Proceedings of the 10th Annual Water Distribution Systems Analysis Conference, WDSA 2008.

[bib8] Adewumi J.R., Ilemobade A.A., Van Zyl J.E. (2010). Treated wastewater reuse in South Africa: overview, potential and challenges. Resour. Conserv. Recycl..

[bib9] Adewumi J.R., Ilemobade A.A., Van Zyl J.E. (2014). Factors predicting the intention to accept treated wastewater reuse for non-potable uses amongst domestic and non-domestic respondents. J. S. Afr. Inst. Civ. Eng..

[bib10] Akpan V.E., Olukanni D.O. (2020). Hazardous waste Management : an African overview. Recycling.

[bib11] Alygizakis N.A., Urík J., Beretsou V.G., Kampouris I., Galani A., Oswaldova M. (2020). Evaluation of chemical and biological contaminants of emerging concern in treated wastewater intended for agricultural reuse. Environ. Int..

[bib13] Baawain M.S., Al-Mamun A., Omidvarborna H., Al-Sabti A., Choudri B.S. (2020). Public perceptions of reusing treated wastewater for urban and industrial applications: challenges and opportunities. Environ. Dev. Sustain..

[bib14] Bailey E.S., Casanova L.M., Simmons O.D., Sobsey M.D. (2018). Tertiary treatment and dual disinfection to improve microbial quality of reclaimed water for potable and non-potable reuse: a case study of facilities in North Carolina. Sci. Total Environ..

[bib15] Bain R.E., Gundry S.W., Wright J.A., Yang H., Pedley S., Bartram J.K. (2012). Accounting for water quality in monitoring access to safe drinking-water as part of the Millennium Development Goals: lessons from five countries. Bull. World Health Organ..

[bib16] Beler-Baykal B. (2015). Stream segregation in household use: a review of grey water as an alternative source of water and yellow water as an alternative source of fertilizers. Water Qual. Expo. Health.

[bib17] Bose D., Dhawan H., Kandpal V., Vijay P., Gopinath M. (2018). Sustainable power generation from sewage and energy recovery from wastewater with variable resistance using microbial fuel cell. Enzym. Microb. Technol..

[bib18] Bungu L.C. (2014). Assessing the Perceptions of Consumers on Wastewater Reuse in the Vaal Triangle LC Bungu 23510994. https://pdfs.semanticscholar.org/cc8c/2f744e5b5ff65464356e6752b2045bdd0f04.pdf?_ga=2.173890595.1632497263.1592747043-895415340.1571778895.

[bib19] Buyukkamaci N., Alkan H.S. (2013). Public acceptance potential for reuse applications in Turkey. Resour. Conserv. Recycl..

[bib20] Capodaglio A.G. (2017). Integrated, decentralized wastewater management for resource recovery in rural and peri-urban areas. Resources.

[bib21] Carr G., Potter R.B., Nortcliff S. (2011). Water reuse for irrigation in Jordan: perceptions of water quality among farmers. Agric. Water Manag..

[bib22] Cheng H., Hu Y., Zhao J. (2009). Meeting China’s water shortage crisis: current practices and challenges. Environ. Sci. Technol..

[bib23] Chianu J.N., Tsujii H., Kormawa P. (2004). Agriculture in the savannas of northern Nigeria. Outlook Agric..

[bib24] Cobbett E. (2018). Gatekeepers of financial power: from London to Lagos. Third World Thematics: A TWQ J..

[bib25] Corcoran E., Nellemann C., Baker E., Bos R., Osborn D., Savelli H. (2010). Sick Water? the Central Role of Wastewater Management in Sustainable Development: A Rapid Response Assessment. http://www.coalition-eau.org/wp-content/uploads/SickWater_screen.pdf.

[bib26] Degefu D.M., Weijun H., Zaiyi L., Liang Y., Zhengwei H., Min A. (2018). Mapping monthly water scarcity in global transboundary basins at country-basin mesh based spatial resolution. Sci. Rep..

[bib27] Delli Compagni R., Gabrielli M., Polesel F., Turolla A., Trapp S., Vezzaro L., Antonelli M. (2020). Risk assessment of contaminants of emerging concern in the context of wastewater reuse for irrigation: an integrated modelling approach. Chemosphere.

[bib28] Doǧruel S., Germirli-Babuna F., Kabdaşli I., Insel G., Orhon D. (2003). Effect of stream segregation on ozonation for the removal of significant COD fractions from textile wastewater. J. Chem. Technol. Biotechnol..

[bib29] Dolnicar S., Schäfer A.I. (2009). Desalinated versus recycled water: public perceptions and profiles of the accepters. J. Environ. Manag..

[bib30] Duke M., Ramchandran L., Anderson R., Gray S. (2020). Firefighting Wastewater Treatment Utilising Powdered Activated Carbon and Ceramic Membranes.

[bib31] FAO (2015). The State of Food Insecurity in the World. Meeting the 2015 International Hunger Targets: Taking Stock of Uneven Progress. http://www.fao.org/3/a-i4646e.pdf%0D.

[bib32] Fielding K.S., Dolnicar S., Schultz T. (2019 July 4). Public acceptance of recycled water. Int. J. Water Resour. Dev..

[bib33] Friedler E., Lahav O. (2006). Centralised urban wastewater reuse: what is the public attitude?. Water Sci. Technol..

[bib34] Friedler E., Lahav O., Jizhaki H., Lahav T. (2006). Study of urban population attitudes towards various wastewater reuse options: Israel as a case study. J. Environ. Manag..

[bib35] Gain A.K., Wada Y. (2014). Assessment of future water scarcity at different spatial and temporal scales of the Brahmaputra river basin. Water Resour. Manag..

[bib36] Ganguli P., Kumar D., Ganguly A.R. (2017). US power production at risk from water stress in a changing climate. Sci. Rep..

[bib37] Garcia-Cuerva L., Berglund E.Z., Binder A.R. (2016). Public perceptions of water shortages, conservation behaviors, and support for water reuse in the U.S.. Resour. Conserv. Recycl..

[bib38] Garcia X., Pargament D. (2015). Reusing wastewater to cope with water scarcity: economic, social and environmental considerations for decision-making. Resour. Conserv. Recycl..

[bib39] Gerba C.P., Choi C.Y. (2009). Water quality. The Produce Contamination Problem.

[bib40] Ghernaout D., Elboughdiri N. (2019). Upgrading wastewater treatment plant to obtain drinking water. OALib.

[bib41] Giagnorio M., Ricceri F., Tiraferri A. (2019). Desalination of brackish groundwater and reuse of wastewater by forward osmosis coupled with nanofiltration for draw solution recovery. Water Res..

[bib42] Gu Q., Chen Y., Pody R., Cheng R., Zheng X., Zhang Z. (2015). Public perception and acceptability toward reclaimed water in Tianjin. Resour. Conserv. Recycl..

[bib43] Hagenvoort J., Ortega-Reig M., Botella S., García C., de Luis A., Palau-Salvador G. (2019). Reusing treated waste-water from a circular economy perspective—the case of the real Acequia de Moncada in Valencia (Spain). Water.

[bib44] Hartley T.W. (2006). Public perception and participation in water reuse. Desalination.

[bib45] Hoekstra A.Y. (2014). April 25). Water scarcity challenges to business. Nat. Clim. Change.

[bib46] Ilesanmi A.O. (2010). Urban sustainability in the context of Lagos mega-city. J. Geogr. Reg. Plann..

[bib47] Isiorho S.A., Oginni F.A. (2014). Assessment of waste water treatment in Canaanland, Ota, Ogun state, N.... https://www.slideshare.net/felixss/assessment-of-waste-water-treatment-in-canaanland-ota-ogun-state-nigeriaoginni-isiorho-paper-1st-postgraduatepostcon2008iupdur.

[bib48] Isiorho S.A., Omole D.O., Ogbiye S.A., Olukanni D.O., Ede A.N., Akinwumi I.I. (2014). Study of reed-bed of an urban wastewater in a Nigerian community. Proceedings of the IASTED International Conference on Environmental Management and Engineering, EME 2014.

[bib49] IWA (2018). Wastewater Report 2018. http://www.iwa-network.org/wp-content/uploads/2018/02/OFID-Wastewater-report-2018.pdf.

[bib50] Jaramillo M.F., Restrepo I. (2017). Wastewater reuse in agriculture: a review about its limitations and benefits. Sustainability (Switzerland).

[bib51] Jeremy (2015). The Many Benefits of Water Recycling - M.W. Watermark. http://www.mwwatermark.com/en_US/many-benefits-water-recycling/.

[bib52] Jiang Y. (2009 August 1). China’s water scarcity. J. Environ. Manag..

[bib53] Jiang Y. (2015 December 1). China’s water security: current status, emerging challenges and future prospects. Environ. Sci. Pol..

[bib54] Kawale H.D., Ranveer A.C., Chavan A.R. (2017). Electricity generation from wastewater using a microbial fuel cell by using mixed bacterial culture. J. Biochem. Technol..

[bib55] Kayode O., Luethi C., Rene E. (2018). Management recommendations for improving decentralized wastewater treatment by the food and beverage industries in Nigeria. Environments.

[bib56] Khan M., Evans A., Chadwick M. (2011). Flow segregation options to reduce effluent treatment plant running costs. International Conference on Chemical Engineering.

[bib57] Khanpae M., Karami E., Maleksaeidi H., Keshavarz M. (2020). Farmers’ attitude towards using treated wastewater for irrigation: the question of sustainability. J. Clean. Prod..

[bib58] Kihila J., Mtei K.M., Njau K.N. (2014). Wastewater treatment for reuse in urban agriculture; the case of Moshi Municipality, Tanzania. Phys. Chem. Earth.

[bib59] Kummu M., Guillaume J.H.A., De Moel H., Eisner S., Flörke M., Porkka M. (2016). The world’s road to water scarcity: shortage and stress in the 20th century and pathways towards sustainability. Sci. Rep..

[bib60] Ladan M.T. (2013). Review of NSREA act 2007 and Regulations 2009-2011: a new dawn in environmental compliance and enforcement in Nigeria. SSRN Electronic J..

[bib61] Ma T., Sun S., Fu G., Hall J.W., Ni Y., He L. (2020). Pollution exacerbates China’s water scarcity and its regional inequality. Nat. Commun..

[bib62] Maleksaeidi H., Ranjbar S., Eskandari F., Jalali M., Keshavarz M. (2018). Vegetable farmers’ knowledge, attitude and drivers regarding untreated wastewater irrigation in developing countries: a case study in Iran. J. Clean. Prod..

[bib63] Mantu J.I. (2019). NESREA and the challenge of enforcing the provisions of environmental impact assessment act in Nigeria. SSRN Electronic J..

[bib64] Marron E.L., Mitch W.A., Gunten U. Von, Sedlak D.L. (2019). A tale of two treatments: the multiple barrier approach to removing chemical contaminants during potable water reuse. Acc. Chem. Res..

[bib65] Mary O. (2014). Availability and use of domestic water in Osiele area of Ogun state, Nigeria. Res. J. Eng. Appl. Sci..

[bib66] Massoud M.A., Kazarian A., Alameddine I., Al-Hindi M. (2018). Factors influencing the reuse of reclaimed water as a management option to augment water supplies. Environ. Monit. Assess..

[bib67] McNeill L.S., Almasri M.N., Mizyed N. (2009). A sustainable approach for reusing treated wastewater in agricultural irrigation in the West Bank - Palestine. Desalination.

[bib68] Moe C.L., Rheingans R.D. (2006). Global challenges in water, sanitation and health. J. Water Health.

[bib69] Mogaji E. (2020). Impact of COVID-19 on transportation in Lagos, Nigeria. Transport. Res. Interdis. Perspect..

[bib70] Mu’azu N.D., Abubakar I.R., Blaisi N.I. (2020). Public acceptability of treated wastewater reuse in Saudi Arabia: implications for water management policy. Sci. Total Environ..

[bib71] Naik S., Jujjavarappu S.E. (2020). Simultaneous bioelectricity generation from cost-effective MFC and water treatment using various wastewater samples. Environ. Sci. Pollut. Control Ser..

[bib72] Nancarrow B.E., Leviston Z., Po M., Porter N.B., Tucker D.I. (2008). What drives communities’ decisions and behaviours in the reuse of wastewater. Water Sci. Technol..

[bib73] Navarro I., Chavez A., Barrios J.A., Maya C., Becerril E., Lucario S., Jimenez B. (2015). Wastewater reuse for irrigation — practices, safe reuse and perspectives. Irrigation and Drainage - Sustainable Strategies and Systems.

[bib74] NESREA (2009 September 30). National Environmental Standards and Regulations Enforcement Agency (Establishment) Act, 2007. https://www.nesrea.gov.ng/wp-content/uploads/2020/02/Sanitation_and_Wastes_Control_Regulations%202009.pdf.

[bib75] NESREA (2020). NESREA Official Website | About Us. https://www.nesrea.gov.ng/about-us/.

[bib76] Nwankwoala H.O. (2011). An Integrated Approach to Sustainable Groundwater Development and Management in Nigeria. http://citeseerx.ist.psu.edu/viewdoc/download?doi=10.1.1.1001.1447&rep=rep1&type=pdf.

[bib77] Ohwo O., Abotutu A. (2014). Access to potable water supply in Nigerian cities evidence from Yenagoa Metropolis. Am. J. Water Res..

[bib78] Omole (2013). Sustainable groundwater exploitation in Nigeria. J. Water Resour. Ocean Sci..

[bib79] Omole, Alade O.O., Emenike P.C., Tenebe I.T., Ogbiye A.S., Ngene B.U., Kingdom W.I.U. (2017). Quality assessment of a university campus wastewater resource.

[bib80] Omole D.O., Ndambuki J.M. (2014). Sustainable living in Africa: case of water, sanitation, air pollution and energy. Sustainability (Switzerland).

[bib81] Omole David O., Ogbiye A.S., Longe E.O., Adewumi I.K., Elemile O.O., Tenebe T.I. (2018). Water quality checks on river atuwara, south-west Nigeria. WIT Trans. Ecol. Environ..

[bib82] Omole, Jim-George T., Akpan V. (2019). Economic analysis of wastewater reuse in Covenant university. J. Phys. Conf..

[bib83] Ormerod K.J., Scott C.A. (2013). Drinking wastewater. Sci. Technol. Hum. Val..

[bib84] Oteng-Peprah M., Acheampong M.A., deVries N.K. (2018). Greywater characteristics, treatment systems, reuse strategies and user perception—a review. Water Air Soil Pollut..

[bib85] Prajapati B. (2018). Decentralized Grey Water Reuse: Developing Grey Water Treatment Technology and Mapping End-Users’ Perceptions.

[bib86] Qadir M., Wichelns D., Raschid-Sally L., McCornick P.G., Drechsel P., Bahri A., Minhas P.S. (2010). The challenges of wastewater irrigation in developing countries. Agric. Water Manag..

[bib87] Redman S., Ormerod K.J., Kelley S. (2019). Reclaiming suburbia: differences in local identity and public perceptions of potable water reuse. Sustainability.

[bib88] Ren L., Xue L., Liu Y., Shi J., Han Q., Yi P. (2017). Study on variations in climatic variables and their influence on runoff in the Manas river basin, China. Water.

[bib89] Ren X., Zhang Y., Chen H. (2019). Graywater treatment technologies and reuse of reclaimed water for toilet flushing. Environ. Sci. Pollut. Control Ser..

[bib90] Ricart S., Rico A.M., Ribas A. (2019). Risk-yuck factor nexus in reclaimed wastewater for irrigation: comparing farmers’ attitudes and public perception. Water.

[bib91] Rice J., Stotts R., Wutich A., White D., Maupin J., Brewis A. (2019). Motivators for treated wastewater acceptance across developed and developing contexts. J. Water, Sanit. Hyg. Dev..

[bib92] Rice J., Wutich A., White D.D., Westerhoff P. (2016). Comparing actual de facto wastewater reuse and its public acceptability: a three city case study. Sustain. Cities Soc..

[bib93] Robinson K.G., Robinson C.H., Hawkins S.A. (2005). Assessment of public perception regarding wastewater reuse. Water Sci. Technol. Water Supply.

[bib94] Rock C., Solop F.I., Gerrity D. (2012). Survey of statewide public perceptions regarding water reuse in Arizona. J. Water Supply Res. Technol. - Aqua.

[bib95] Rodriguez Boluarte I.A., Andersen M., Pramanik B.K., Chang C.Y., Bagshaw S., Farago L. (2016). Reuse of car wash wastewater by chemical coagulation and membrane bioreactor treatment processes. Int. Biodeterior. Biodegrad..

[bib96] Ross V.L., Fielding K.S., Louis W.R. (2014). Social trust, risk perceptions and public acceptance of recycled water: testing a social-psychological model. J. Environ. Manag..

[bib97] Saliba R., Callieris R., D’Agostino D., Roma R., Scardigno A. (2018). Stakeholders’ attitude towards the reuse of treated wastewater for irrigation in Mediterranean agriculture. Agric. Water Manag..

[bib98] Schwabe K., Nemati M., Amin R., Tran Q., Jassby D. (2020). Unintended consequences of water conservation on the use of treated municipal wastewater. Nat. Sustain..

[bib99] Sgroi M., Vagliasindi F.G.A., Roccaro P. (2018 April 1). Feasibility, sustainability and circular economy concepts in water reuse. Curr. Opin. Environ. Sci. Health.

[bib100] Shakir E., Zahraw Z., Al-Obaidy A.H.M.J. (2017). Environmental and health risks associated with reuse of wastewater for irrigation. Egypt. J. Pet..

[bib101] Smith H.M., Brouwer S., Jeffrey P., Frijns J. (2018). February 1). Public responses to water reuse – understanding the evidence. J. Environ. Manag..

[bib102] SSWM (2020). Wastewater Reuse in Industry | SSWM - Find Tools for Sustainable Sanitation and Water Management!. https://sswm.info/water-nutrient-cycle/water-use/hardwares/optimisation-water-use-industries/wastewater-reuse-in-industry.

[bib103] Taemthong W. (2018). Grey water recycling for reuse in toilet flushing: a case study in Thailand. J. Green Build..

[bib104] Tatinclaux M., Gregoire K., Leininger A., Biffinger J.C., Tender L., Ramirez M. (2018). Electricity generation from wastewater using a floating air cathode microbial fuel cell. Water-Energy Nexus.

[bib105] Tortajada C. (2020). Contributions of recycled wastewater to clean water and sanitation Sustainable Development Goals. NPJ Clean Water.

[bib106] Uçar D. (2018). Membrane processes for the reuse of car washing wastewater. J. Water Reuse Desal..

[bib107] UCLA (2019). What Does Cronbach’s Alpha Mean? | SPSS FAQ. https://stats.idre.ucla.edu/spss/faq/what-does-cronbachs-alpha-mean/.

[bib108] UN-HABITAT (2014). State of the World’s Cities 2012/2013 Prosperity of Cities.

[bib109] UN-Women (2020). Facts & Figures | UN Women – Headquarters. https://www.unwomen.org/en/news/in-focus/commission-on-the-status-of-women-2012/facts-and-figures.

[bib110] United Nations (2020). THE 17 GOALS | Department of Economic and Social Affairs. https://sdgs.un.org/goals.

[bib111] United Nations University (2019). UN: Rising Reuse of Wastewater in Forecast but World Lacks Data on “Massive Potential Resource” - United Nations University. https://unu.edu/media-relations/releases/rising-reuse-of-wastewater-in-forecast-but-world-lacks-data.html.

[bib112] Wei X., Binger Z.M., Achilli A., Sanders K.T., Childress A.E. (2020). A modeling framework to evaluate blending of seawater and treated wastewater streams for synergistic desalination and potable reuse. Water Res..

[bib113] Wester J., Timpano K.R., Çek D., Lieberman D., Fieldstone S.C., Broad K. (2015). Psychological and social factors associated with wastewater reuse emotional discomfort. J. Environ. Psychol..

[bib114] Wilson Z., Pfaff B. (2008). Religious, philosophical and environmentalist perspectives on potable wastewater reuse in Durban, South Africa. Desalination.

[bib115] World Bank (2019 July 1). New Country Classifications by Income Level: 2019-2020. https://blogs.worldbank.org/opendata/new-country-classifications-income-level-2019-2020.

[bib116] Yue W., Cai Y., Xu L., Yang Z., Yin X., Su M. (2017). Industrial water resources management based on violation risk analysis of the total allowable target on wastewater discharge. Sci. Rep..

[bib117] Zabala J.A., Dolores de Miguel M., Martínez-Paz J.M., Alcon F. (2019). Perception welfare assessment of water reuse in competitive categories. Water Sci. Technol. Water Supply.

