# Supplementary material for: Assessing the public perceptions of treated wastewater reuse: opportunities and implications for urban communities in developing countries
Source: Heliyon. 2020 Oct 14;6(10):e05246. doi: 10.1016/j.heliyon.2020.e05246 (PMC7556266; doi:10.1016/j.heliyon.2020.e05246)
Supplement: Supplementary Material [file mmc1.pdf]

Assessing the Public Perception of Treated Wastewater Reuse in Canaanland, Nigeria

This questionnaire is a research tool designed to determine the perception of the Canaanland city on the subject of treated Wastewater Reuse.
*Reclaimed or recycled water (also called wastewater reuse or water reclamation) is the process of converting wastewater into water that can be reused for other purposes.

* Required

1. Gender *

Mark only one oval.

- Male
- Female

2. Age Group *

Mark only one oval.

- 16-21
- 21-30
- 31-40
- 41-50
- 51-60
- >60

3. Race *

Mark only one oval.

- Black
- Coloured
- White
- Indian
- Other: ____________________

4. Level of Employment *

Mark only one oval.

- Employed
- Self Employed
- Unemployed
- Student

5. Educational Qualifications *

Mark only one oval.

- No level of Education
- Secondary School Certificate
- Diploma (OND/HND)
- Undergraduate Student (100 to 500 level)
- Graduate (BSc)
- Masters Degree
- PhD

6. Residence

Mark only one oval.

- Within Canaanland or Covenant University
- Ota and Environs
- Other: ____________________

7. Knowledge on Global Water Shortage *

Mark only one oval.

- No idea at all
- Little knowledge
- Sufficient Knowledge
- Highly Knowledgeable

8. What is your knowledge on Wastewater Recycling and Reuse? *

Mark only one oval.

- No idea at all
- Little knowledge
- Sufficient Knowledge
- Highly Knowledgeable

9. Have you ever seen a wastewater treatment facility?

Mark only one oval.

- Yes
- No
- Maybe

10. Recycling & reusing wastewater is environmentally responsible *

Mark only one oval.

- Strongly disagree
- Disagree
- Neutral
- Agree
- Strongly agree

11. Recycling & Reusing wastewater protects the environment from pollutants

Mark only one oval.

- Strongly disagree
- Disagree
- Neutral
- Agree
- Strongly agree

12. Recycling & Reusing Wastewater could cause health concerns *

Mark only one oval.

- Strongly disagree
- Disagree
- Neutral
- Agree
- Strongly agree

13. Reusing wastewater will reduce the need for water treatment plant expansions

Mark only one oval.

- Strongly disagree
- Disagree
- Neutral
- Agree
- Strongly agree

14. Recycling & Reusing wastewater will bring about economic benefits

Mark only one oval.

- Strongly disagree
- Disagree
- Neutral
- Agree
- Strongly agree

15. Have you reused wastewater domestically?

Mark only one oval.

- Frequently
- When there is no water supply
- Not very often
- Never (I can't stand the idea)

16. I will be willing to use treated wastewater for the following if proper planning was put into it *

Check all that apply.

- [ ] Industrial Use
- [ ] Firefighting
- [ ] Washing Cars
- [ ] Washing cloths
- [ ] Watering vegetable gardens
- [ ] Watering lawns
- [ ] Cooking food
- [ ] Flushing toilets
- [ ] Swimming pools
- [ ] Drinking
- [ ] Generate Electricity
- [ ] Construction of Buildings

17. I have concerns about using treated wastewater because of? *

Check all that apply.

- [ ] Health reasons
- [ ] Psychological reasons
- [ ] Religious reasons
- [ ] I do not trust the treatment process
- [ ] Mechanical or equipment breakdown
- [ ] Quality of the water
- [ ] Lack of adequate revenues to fund the process
- [ ] Poor Management of the plant

18. I get information about the environment via the following sources

Check all that apply.

- [ ] Newspaper & magazines
- [ ] Television & Radios
- [ ] Internet Sources
- [ ] Environmental groups & NGO’s
- [ ] Academic Research
- [ ] Municipal authorities
- [ ] Universities
- [ ] Never used any of the above

19. Does your institution Or community have any Wastewater treatment facility?

Mark only one oval.

- ( ) Yes
- ( ) No
- ( ) Maybe

20. I will trust wastewater reuse processes if it is endorsed by

Check all that apply.

- [ ] Local Municipalities
- [ ] State and Federal Government
- [ ] Medical Doctors
- [ ] University Professors & Experts
- [ ] Farmers or Industrialist
- [ ] Internet & media sources

21. I would like wastewater to be recycled at the following levels

Check all that apply.

- [ ] Own household
- [ ] Entire campus
- [ ] Whole city

22. What wastewater source can be recycled?

Check all that apply.

- [ ] Rainwater from my roof to augment water supply
- [ ] I prefer to use greywater (water from washing dishes, cloths, own body) to augment water supply
- [ ] I prefer to use wastewater from treatment plants to augment water supply

23. Do you believe money can be made from wastewater reuse?

Mark only one oval.

- ◯ Yes
- ◯ No
- ◯ Maybe

24. Recycled wastewater can serve as a source of Fertilizer

Mark only one oval.

- ◯ Strongly disagree
- ◯ Disagree
- ◯ Neutral
- ◯ Agree
- ◯ Strongly agree

25. Wastewater reused in agriculture can boost agricultural yield

Mark only one oval.

- Strongly disagree
- Disagree
- Neutral
- Agree
- Strongly agree

26. Energy saving is a potential of wastewater reuse

Mark only one oval.

- Strongly Disagree
- Disagree
- Neutral
- Agree
- Strongly agree

27. Do you think groundwater levels in your community is sustainable at the current pumping rate?

Mark only one oval.

- Yes
- No
- Maybe

28. State any comments you have on wastewater reuse

This content is neither created nor endorsed by Google.

Google Forms
